# The prognostic impact of systemic inflammation and nutritional indicators on targeted therapy for renal cell carcinoma: a systematic review and meta-analysis

**DOI:** 10.3389/fnut.2026.1777753

**Published:** 2026-02-25

**Authors:** Yushuang Xiao, Yang Dai, Xushu Zhong, Luocheng Zhang, Ruilian Xie, Qiaolin Zhou, Wenyi Liang, Xu Sun, Mengting Yang, Zhuohang Zou, Yutong Wang, He Li, Ting Niu

**Affiliations:** 1 Department of Hematology, Institute of Hematology, and Center for High Altitude Medicine, West China Hospital, Sichuan University, Chengdu, China; 2 West China Medical School, West China Hospital, Sichuan University, Chengdu, China; 3 State Key Laboratory of Biotherapy, Collaborative Innovation Center of Biotherapy, West China Hospital, Sichuan University, Chengdu, China; 4 National Facility for Translational Medicine (Sichuan), West China Hospital, Sichuan University, Chengdu, China

**Keywords:** inflammatory indicators, nutritional indicators, prognosis, renal cell carcinoma, targeted therapy

## Abstract

**Introduction:**

The prognosis of metastatic renal cell carcinoma (mRCC) varies significantly, and early treatment failure is common. Systemic inflammation and nutritional indicators may predict the survival outcomes of patients with renal cell carcinoma (RCC) receiving targeted therapy. However, an integrated evaluation of the prognostic value of these markers is still lacking. This study aims to comprehensively assess and compare the prognostic significance of various systemic inflammation and nutritional indicators for the overall survival (OS) and progression-free survival (PFS) of patients with RCC undergoing targeted therapy through systematic review and meta-analysis.

**Methods:**

According to PRISMA guidelines, a systematic search was performed across PubMed, Embase, Cochrane Library, Web of Science, Scopus, Medline up to October 17, 2025. Retrospective cohort studies evaluating the correlation between baseline inflammatory/nutritional indicators and OS/PFS in RCC patients receiving targeted therapy were included. And biomarkers included Neutrophil-to-Lymphocyte Ratio (NLR), C-Reactive Protein (CRP), Platelet-to-Lymphocyte Ratio (PLR), Body Mass Index (BMI), Albumin (Alb), Prognostic Nutritional Index (PNI), Systemic Immune-Inflammation Index (SII), Neutrophil Count (NEU), and hemoglobin (Hb). Two researchers independently performed literature screening, data extraction, and quality assessment. Meta-analysis was performed using Stata 18.0. The pooled hazard ratio (HR) and its 95% confidence interval (CI) were calculated. And heterogeneity tests, sensitivity analyses, subgroup analyses, and publication bias assessments were conducted. This analysis has been registered in PROSPERO (CRD420251167462).

**Results:**

A total of 152 retrospective cohort studies involving 33,842 patients were included. The meta-analysis revealed that high baseline NLR, NEU, SII, and low Hb levels were significantly associated with poorer OS. The combined HR values were as follows: NLR for OS: HR = 1.82, 95% CI = 1.67–1.99; NEU for OS: HR = 1.88, 95% CI = 1.67–2.12; SII for OS: HR = 1.92, 95% CI = 1.72–2.15; Hb for OS: HR = 1.74, 95% CI = 1.58–1.92. Additionally, NLR (HR = 1.51, 95% CI = 1.38–1.65) was also significantly associated with poorer PFS.

**Discussion:**

NLR, NEU, SII and Hb are robust and consistent prognostic predictors for patients with RCC receiving targeted therapy. The evidence all stems from retrospective observational studies therefore selection bias and the influence of unmeasured confounding factors could not be fully controlled for.

**Systematic review registration:**

https://www.crd.york.ac.uk/PROSPERO/view/CRD420251167462, PROSPERO: CRD420251167462.

## 1 Introduction

RCC is a malignant tumor originating from the epithelial cells of the renal tubules. As a major type of cancer in the urinary system, it accounts for approximately 2.2% of all cancers with over 400,000 new cases and more than 150,000 deaths in 2022 (1). Among these cases, mRCC is a highly invasive type with poor prognosis, and the 5-year survival rate is usually less than 20% (2). The treatment of mRCC has been continuously evolving in recent years, transitioning from the initial surgeries, radiotherapy, and chemotherapy to targeted therapy and immunotherapy. The systemic treatment using tyrosine kinase inhibitors (TKIs) and immune checkpoint inhibitors (ICIs) has been established as the standard treatment and common regimens include axitinib with pembrolizumab, cabozantinib with nivolumab, and ipilimumab with nivolumab and so on (3).

Despite the advancements in treatment, the significant differences in mRCC patients' prognosis outcomes remain a major challenge and early treatment failure is common (4). Common and easily accessible biomarkers, such as genomic, immunologic, and radiologic biomarkers, may predict treatment failure in specific therapeutic contexts (5, 6). Specifically, various laboratory values and blood count parameters, including inflammatory response and nutritional status, have been proposed as independent prognostic factors for mRCC (7–9). These markers can be categorized into three main groups: single inflammatory indicators (e.g., CRP, NEU); single nutritional indicators (e.g., BMI, Alb, Hb); and inflammatory-nutritional composite indexes (e.g., NLR, PLR, SII, PNI). All have demonstrated prognostic value in the targeted treatment of mRCC (10–16). However, most of the current prognosis studies are limited to small sample sizes, and the reliability of the evidence still requires validation through larger-scale clinical studies.

Therefore, we plan to conduct a systematic review and meta-analysis to comprehensively evaluate and compare the prognostic values of various systemic inflammatory markers and nutritional markers in patients with RCC receiving targeted therapy. Our study aims to integrate existing evidence, systematically evaluate the predictive capabilities of these biomarkers for OS and PFS, and through indirect comparison, identify the most robust and consistent prognostic indicators. This research will help provide evidence-based guidance for the selection of risk stratification tools in clinical practice, lay the foundation for developing individualized prognostic models in the future, and ultimately contribute to improving the treatment decisions and prognosis management for patients with metastatic renal cell carcinoma.

## 2 Methods

### 2.1 Search strategy

We conducted a comprehensive systematic literature search in accordance with the Preferred Reporting Items for Systematic Reviews and Meta-Analyses (PRISMA) guidelines (17), shown in Supplementary Table 1. The search was performed across six major electronic databases: PubMed, Embase, Cochrane Library, Web of Science Core Collection, Scopus, and Medline, from inception until October 17, 2025. The search strategy was designed to include three core conceptual groups: “renal cell carcinoma” AND “inflammatory and nutritional indices” AND “targeted therapy” using a combination of Medical Subject Headings (MeSH) terms and free-text keywords searched in the title and abstract. The complete search strategies for each database are shown in Supplementary Table 2.

### 2.2 Selection criteria

Studies were included if they met the following criteria:

(1) Population: patients of any gender aged≥ 18 years with RCC treated with systemic targeted therapy. This includes both direct molecular-targeted agents, such as vascular endothelial growth factor receptor-tyrosine kinase inhibitors (VEGFR-TKIs) and mammalian target of rapamycin inhibitors (mTOR inhibitors), as well as immune-targeted agents (i.e., immune checkpoint inhibitors, ICIs), which exert antitumor effects through indirect, immune-mediated mechanisms. Treatments could be administered in either the first-line or subsequent settings.;
(2) Intervention and comparison: comparisons between patients with high levels of pretreatment/baseline inflammatory biomarkers or nutritional assessments and those with low levels based on a cutoff value, and studies that regarded those indicators as continuous variables for comparisons;
(3) Study design: full-text publications of original randomized controlled trials (RCTs) or cohort studies;
(4) Studies reporting at least one time-to-event endpoint: OS and/or PFS;
(5) Studies that provided hazard ratios (HRs) with 95% confidence intervals (CIs) directly or with sufficient data for indirect calculations;
(6) English language articles.

We included only the most relevant studies with the most complete outcomes if several articles were based on the same patient material or reported overlapping cohort. In addition, we excluded studies that were meta-analyses, reviews, letters, case reports, animal studies, comments, or conference abstracts. We also excluded studies with insufficient information. Two investigators independently performed the study search, selection, and data extraction, and disagreements were reevaluated by a third investigator.

These criteria were designed to ensure clinical homogeneity and relevance to current adult RCC management. First, patients under 18 years were excluded because pediatric and adult RCC represent fundamentally distinct diseases in clinical practice. While adult RCC is predominantly clear-cell carcinoma driven by VHL mutations and treated with the targeted therapies analyzed in this study, pediatric RCC is primarily translocation-associated (e.g., TFE3 fusions), shows poor response to conventional targeted agents, and relies on surgery and chemotherapy as cornerstone treatments. Second, only studies reporting targeted therapy outcomes were included to maintain focus on the specific therapeutic context under investigation.

Besides, our analysis focused exclusively on OS and PFS because preliminary screening revealed that other endpoints (e.g., objective response rate, disease-free survival) were reported too infrequently and heterogeneously across studies to permit meaningful quantitative synthesis. OS and PFS represented the only consistently reported time-to-event outcomes with sufficient data for robust meta-analysis.

### 2.3 Data extraction and quality assessment

The following items were collected from each study: first author, year of publication, title, study design, histology, sample size, inclusion criteria, type of targeted therapy, and its dose, characteristics of the population, systematic inflammatory or nutritional indicators and their cutoff value, clinical survival endpoints, multivariable-adjusted HR with 95% CI, and the source of HR. The primary endpoint was OS and the secondary endpoint was PFS. We extracted HRs and 95% CIs from Kaplan-Meier curves using previously published methods when multivariable HRs and 95% CIs were not provided (18).

The outcome indicator OS was defined as the time from initiation of targeted therapy to death due to any cause. PFS was defined as the time from initiation of targeted therapy to disease progression (clinical or radiographic) or death from any cause. NLR was calculated as Neutrophil count/Lymphocyte count. SII was calculated as Platelet count × Neutrophil count/Lymphocyte count. PLR was calculated as Platelet count/Lymphocyte count. BMI was calculated as Weight (kg)/Height (m^2^). PNI was calculated as Serum albumin (g/L) + 5 × Total peripheral blood lymphocyte count (× 10^9^/L).

We assessed study quality according to a widely used tool. We used the Newcastle-Ottawa Quality Assessment Scale (NOS) to evaluate the quality of retrospective and prospective cohort studies (19). The scale comprises nine points in total in three parts, including participant selection (0–4 points), comparability (0–2 points), and exposure or outcome assessment (0–3 points). Scores ≥7 indicate high quality.

### 2.4 Statistical analysis

We analyzed the impacts of Alb, BMI, CRP, Hb, NEU, NLR, PLR, PNI, SII and other indicators on OS and/or PFS of RCC patients treated with targeted therapy. We performed meta-analyses to pool the HRs and 95% CIs of OS and PFS using Stata 18.0 College Station, TX, United States with a two-sided significance level of *p* < 0.05. A formal quantitative meta-analysis for a specific biomarker (e.g., NLR) and outcome (OS or PFS) was conducted only if five or more independent studies provided extractable, adjusted hazard ratios for that combination. This pre-specified minimum study threshold was implemented to ensure the robustness and reliability of pooled estimates and the assessment of heterogeneity. Consequently, the scope of the synthesized results for each biomarker was determined by the volume of available published data meeting this criterion. Cochran's *Q* test and *I*^2^ statistics were calculated to assess the degree of heterogeneity across studies, with *I*^2^ > 50% or *p* < 0.05 reflecting significant heterogeneity. A random-effects model was used when heterogeneity was significant; otherwise, we chose a fixed-effects model. To examine the origin of heterogeneity, we performed subgroup analyses on the basis of cutoff value, types of targeted treatment and histology, and used Galbraith (radial) plots as well. We conducted sensitivity analyses using a leave-one-out method to estimate the influence of a single article on the whole analysis. Finally, we applied the Begg's test, Egger's test, and visual inspection of Begg's funnel plots to investigate the risk of publication bias using a significance level of *p* < 0.05. This meta-analysis has been registered in PROSPERO (Registration Number: CRD420251167462).

## 3 Results

### 3.1 Literature search and study selection

**Figure 1:**
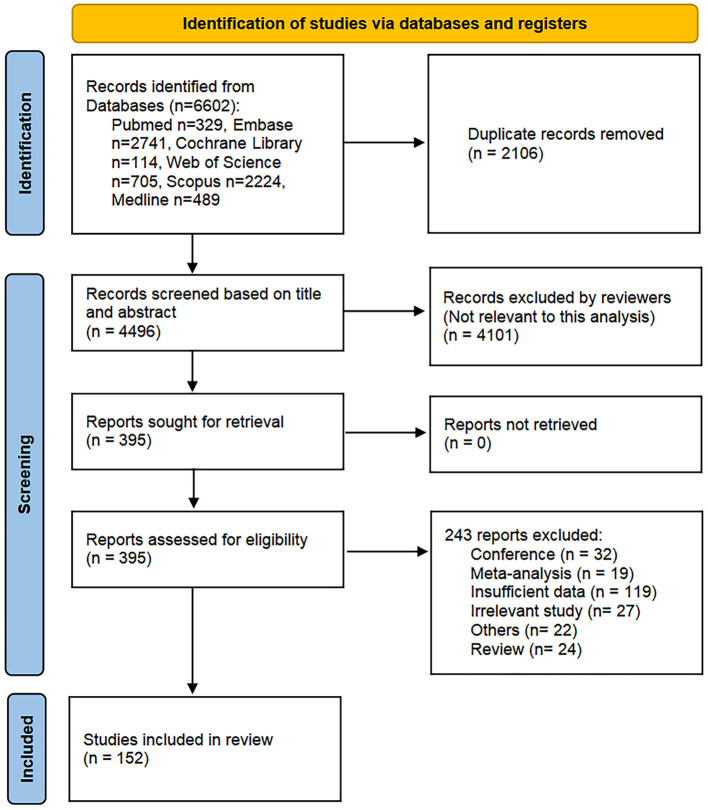
Literature search and study selection results. PRISMA 2020 flow diagram detailing the identification, screening, eligibility assessment, and final inclusion of studies for the systematic review and meta-analysis. The initial search of six databases yielded 6,602 records. After removal of duplicates, 4,496 records were screened by title and abstract. Following exclusion of irrelevant studies, the full texts of 395 articles were assessed for eligibility, resulting in the final inclusion of 152 retrospective cohort studies. The specific reasons for exclusion at the full-text stage are listed in the corresponding box. PRISMA, preferred reporting items for systematic reviews and meta-analyses.

Our literature search and study selection results are shown in Figure 1. We identified 6,602 articles from six databases based on similar searching strategies. After removing 2,106 duplicate records, we screened 4,496 based on title and abstract and then excluded 4,101 articles not completely relevant to this analysis. Finally, we read full texts of 395 articles and included 152 studies. All the studies were retrospective cohort studies. We analyzed 35 studies about NLR, 24 studies about CRP, 10 studies about PLR, 14 studies about BMI, eight studies about Alb, six studies about PNI, eight about SII, 17 studies about NEU, and 18 studies about Hb. Each article can have one or more indicators related to nutrition or inflammation. The indicators involving no more than five articles were not analyzed statistically due to the small sample size. And the first author, publication year, treatment, tumor histology, cutoff value if categorized variables, median OS and PFS with respective HR and 95% CI of analyzed data can be found in Supplementary Table 3.

The characteristics of included studies are as follows (Supplementary Table 4): In total, the studies involved 33,842 patients, with sample sizes ranging from 38 to 1,975 patients. Most studies (*n* = 139) focused on mRCC, while 13 studies focused on advanced renal cell carcinoma (aRCC). Moreover, the clear cell type of RCC accounted for the largest number. Besides, when there were more than one subgroup or cohort in one study, if they, respectively met the inclusion criteria, we would treat them as independent cohorts in statistically combining effect size, like the research conducted by Wang et al. (20). Included studies typically focused on one or two main indicators but also assessed other indicators. We fully extracted the data if it met the inclusion criteria and could be obtained through contact details in the article or by email.

All 152 retrospective cohort studies have been assessed using the NOS tool and results are shown in Supplementary Table 5. Approximately 97% of the studies had a score ≥7; only five studies were scored as 6, indicating that most of the included studies were of high quality.

### 3.2 Association between systemic inflammatory markers level and clinical outcome

NLR, CRP, PLR, BMI, Alb, PNI, SII, NEU, and Hb are systemic inflammation and nutritional indicators associated with clinical outcomes of RCC patients treated with targeted therapy. Most of these markers were categorized into high and low levels based on cutoff values from previous studies or Receiver Operating Characteristic (ROC) curves. And a high baseline NLR, CRP, PLR, SII, NEU, and a low pretreatment BMI, Alb, PNI and Hb were connected with shorter median OS and/or PFS with obvious statistical significance (10–16) (Supplementary Table 3). Besides them, unanalyzed indicators due to insufficient studies also showed some prognostic values: NER, ALC, SMI, TFI, VFA, mGPS (21–26).

The cutoffs of NLR, CRP, PLR, BMI, Alb, PNI, SII, NEU, and Hb were 2.2–5.5, 0.3–1 mg/dl, 144.4–206.9, 18.5–25, 2–4 g/dl, 41–51.62, 730–1,375 (E+9), 4.3–7.5 (E+9/L), 11.5–13 g/dl, respectively according to ROC curve or previous studies. Most of the articles treated the indicators as binary variables. Only a few reported continuous variables, but the samples were small and the data, no analysis was conducted for continuous variables.

### 3.3 Association between high NLR and clinical outcomes

**Figure 2:**
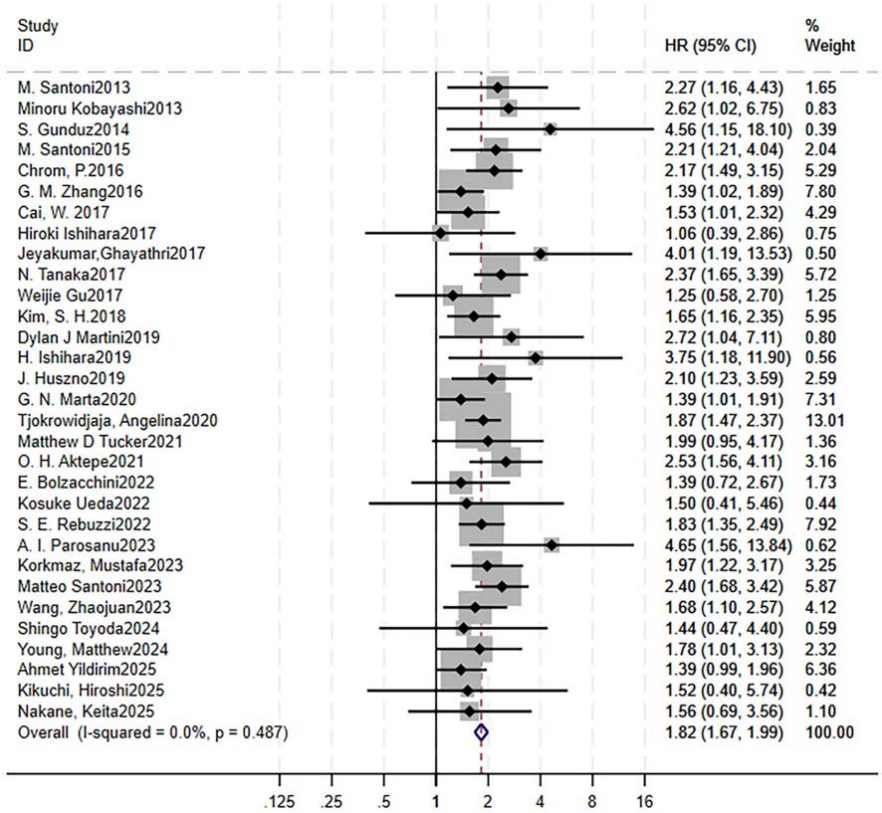
Elevated pretreatment NLR is associated with worse OS in RCC patients receiving targeted therapy. Forest plot showing the pooled HR for the association between high baseline NLR and OS. Square size corresponds to study weight in the meta-analysis. The diamond represents the pooled HR and 95% CI. The vertical dashed line indicates the null effect (HR = 1). Pooled HR = 1.82, 95% CI = 1.67–1.99, *p* < 0.001. Heterogeneity: *I*^2^ = 0%, *p* = 0.49. NLR, neutrophil-to-lymphocyte ratio; OS, overall survival; RCC, renal cell lymphoma; HR, hazard ratio.

**Figure 3:**
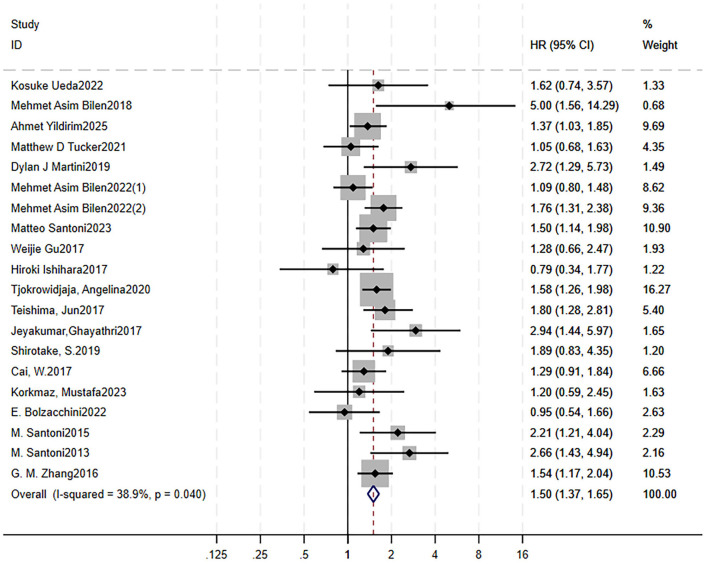
High pretreatment NLR is associated with worse PFS in RCC patients receiving targeted therapy. Forest plot showing the pooled HR for the association between elevated baseline NLR and PFS. Each study is represented by a square (point estimate) and horizontal line (95% CI). Square size corresponds to study weight in the meta-analysis. The diamond represents the pooled HR and 95% CI. The vertical dashed line indicates the null effect (HR = 1). Pooled HR = 1.51, 95% CI = 1.38–1.65, *p* < 0.001. Heterogeneity: *I*^2^ = 38.9%, *p* = 0.04. NLR, neutrophil-to-lymphocyte ratio; PFS, progression free survival; RCC, renal cell lymphoma; HR, hazard ratio.

A total of 35 studies were included to analyze the impact of high NLR on clinical outcomes: 31 on OS (15, 16, 21, 22, 27–53) and 19 on PFS (15, 16, 21, 22, 27, 28, 31, 32, 35, 36, 41–44, 51, 54–57). Forest plots showed that RCC patients treated with targeted therapy and high NLR had shorter OS (HR = 1.82, 95% CI = 1.67–1.99, *p* < 0.00001, no heterogeneity, *I*^2^ = 0%, *p* = 0.49) and shorter PFS (HR = 1.51, 95% CI = 1.38–1.65, *p* < 0.00001, moderate heterogeneity, *I*^2^ = 38.9%, *p* = 0.04; Figure 2 for OS, Figure 3 for PFS). Sensitivity analysis using the leave-one-out method showed that the pooling effect size of OS and PFS would not significantly change due to variations of a single study (Supplementary Figures 1a for OS, 1b for PFS).

To investigate the heterogeneity, we analyzed the results of the sensitivity results and found that two studies had a relatively great influence on the pooling effect size, although the HR and CI were overall stable. Moreover, a Galbraith plot also indicated that these two studies might be potential sources of heterogeneity in the association between high NLR and PFS (Supplementary Figure 1c). Thus, we excluded these studies, and the forest plot showed *I*^2^ decreased to 33.9% with a *Q*-test *p*-value of 0.08, indicating insignificant heterogeneity.

Begg's funnel plots were generated to visually assess the potential for publication bias (Supplementary Figures 1d for OS, 1e for PFS). Both Begg's test (OS: 0.122, PFS: 0.218) and Egger's test (OS: 0.114, PFS: 0.284) indicated no significant evidence of publication bias.

### 3.4 Association between high NEU and overall survival

**Figure 4:**
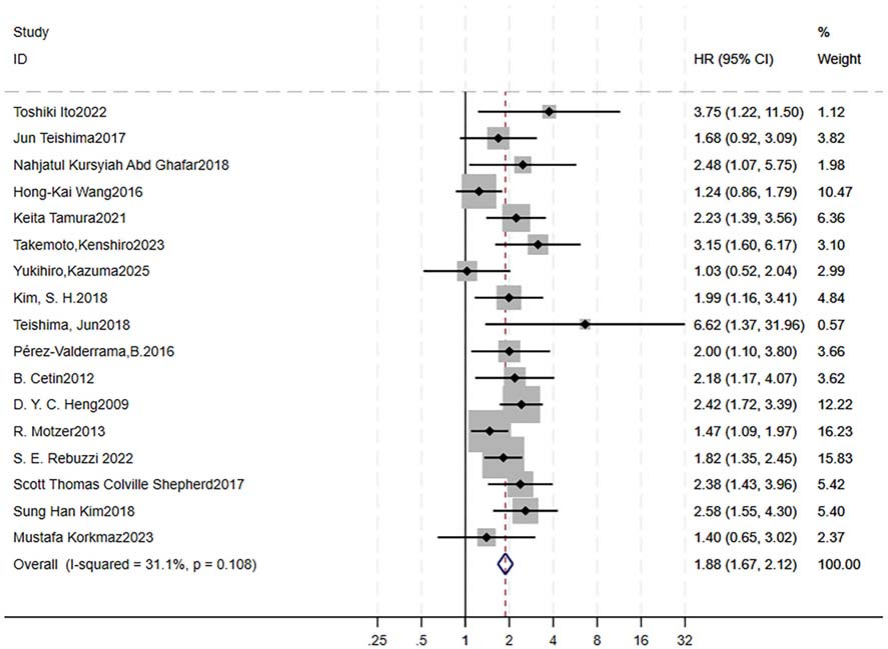
High pretreatment NEU is associated with worse OS in RCC patients receiving targeted therapy. Forest plot showing the pooled HR for the association between elevated baseline NEU and OS. Each study is represented by a square (point estimate) and horizontal line (95% CI). Square size corresponds to study weight in the meta-analysis. The diamond represents the pooled HR and 95% CI. The vertical dashed line indicates the null effect (HR = 1). Pooled HR = 1.88, 95% CI = 1.67–2.12, *p* < 0.001. Heterogeneity: *I*^2^ = 31.1%, *p* = 0.108. OS, overall survival; RCC, renal cell carcinoma; HR, hazard ratio; CI, confidence interval; NEU, neutrophil count.

**Figure 5:**
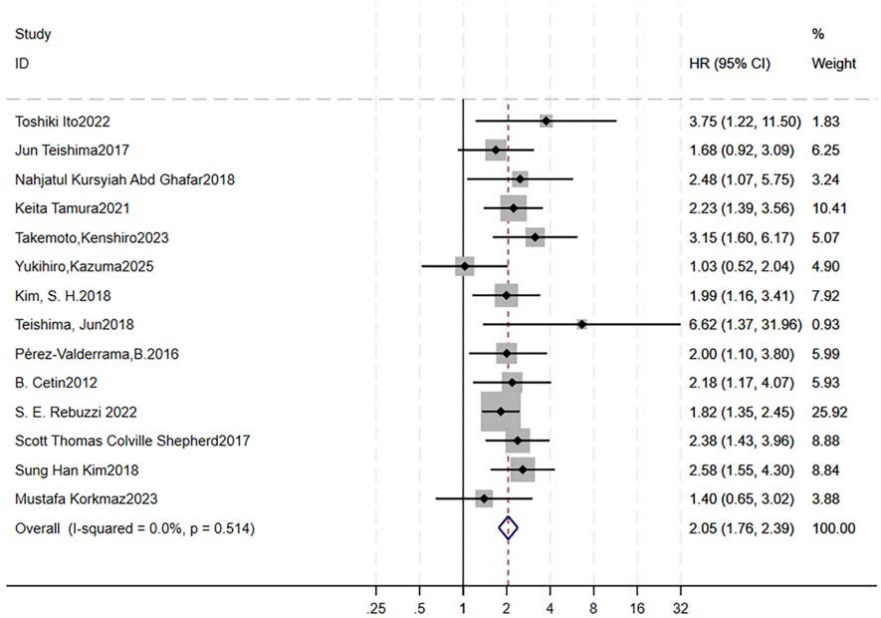
High pretreatment NEU is associated with worse OS in RCC patients receiving targeted therapy after exclusion of potential heterogeneous studies. Forest plot showing the pooled HR for the association between elevated baseline NEU and OS following removal of three studies identified as potential sources of heterogeneity. Each study is represented by a square (point estimate) and horizontal line (95% CI). Square size corresponds to study weight in the meta-analysis. The diamond represents the pooled HR and 95% CI. The vertical dashed line indicates the null effect (HR = 1). Pooled HR = 2.05, 95% CI = 1.76–2.39, *p* < 0.001. Heterogeneity: *I*^2^ = 0%, *p* = 0.514. OS, overall survival; RCC, renal cell carcinoma; HR, hazard ratio; CI, confidence interval; NEU, neutrophil count.

A total of 17 studies were included to analyze the impact of high NEU on OS (9–11, 14, 37, 45, 58–68). We did not analyze its effect on PFS, as only one article involved it. Forest plots showed RCC patients treated with targeted therapy with high NEU had shorter OS (HR = 1.88, 95% CI = 1.67–2.12, *p* < 0.0001; low heterogeneity, *I*^2^ = 31.1 %, *p* = 0.108; Figure 4). Sensitivity analysis using the leave-one-out method showed that the pooling effect size of OS would not significantly change due to variations of a single study; however, three studies indicated relative great impact on it although the HR and CI were overall stable (Supplementary Figure 2a). A Galbraith plot indicated that these three studies could be potential sources of heterogeneity (Supplementary Figure 2b). After excluding them, the forest plot showed *I*^2^ = 0%, with a Q-test *p*-value of 0.514 (Figure 5).

Begg's funnel plots were generated to visually assess the potential for publication bias (Supplementary Figure 2c). Both Begg's test (*p* = 0.249) and Egger's test (*p* = 0.097) indicated no significant evidence of publication bias.

### 3.5 Association between low Hb level and overall survival

**Figure 6:**
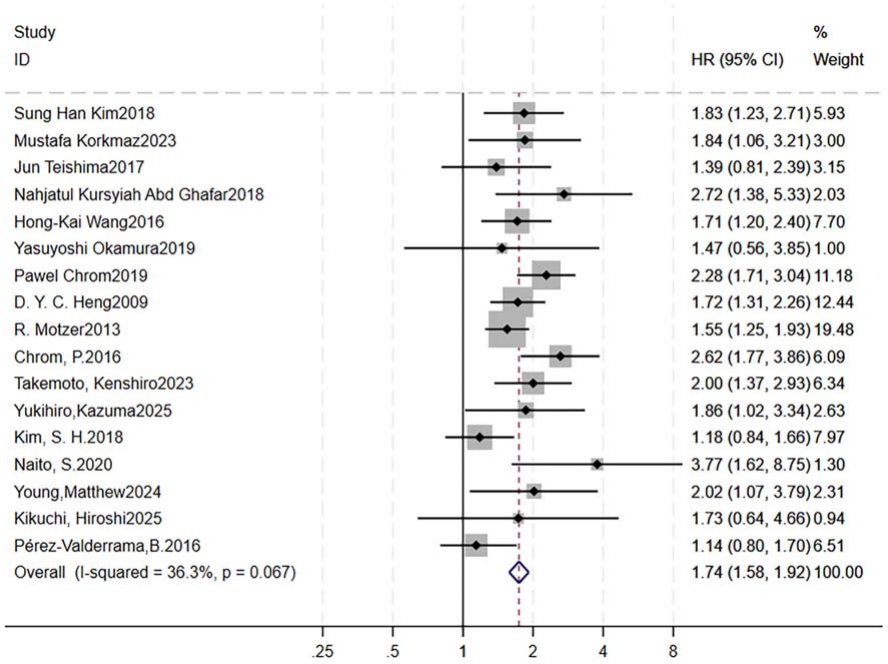
Low pretreatment Hb level is associated with worse OS in RCC patients receiving targeted therapy. Forest plot showing the pooled HR for the association between low baseline Hb and OS. Each study is represented by a square (point estimate) and horizontal line (95% CI). Square size corresponds to study weight in the meta-analysis. The diamond represents the pooled HR and 95% CI. The vertical dashed line indicates the null effect (HR = 1). Pooled HR = 1.74, 95% CI = 1.58–1.92, *p* < 0.001. Heterogeneity: *I*^2^ = 36.3%, *p* = 0.067. OS, overall survival; RCC, renal cell carcinoma; HR, hazard ratio; CI, confidence interval; Hb, hemoglobin.

A total of 17 studies were included to analyze the impact of low hemoglobin level on OS (9, 14, 33, 37, 38, 53, 58–61, 63, 66–71). We did not analyze it for PFS, as only four articles involved it. Forest plots showed RCC patients treated with targeted therapy with low hemoglobin level had shorter OS (HR = 1.74, 95 %CI = 1.58–1.92, *p* < 0.0001; low heterogeneity, *I*^2^ = 36.3 %, *p* = 0.067; Figure 6). Sensitivity analysis using the leave-one-out method showed that the pooling effect size of OS would not significantly change due to variations of a single study (Supplementary Figure 3a). To search for the heterogeneity, a Galbraith plot showed above four studies may be potential sources of heterogeneity (Supplementary Figure 3b). Thus, after excluding them, the forest plot showed *I*^2^ = 0%, with a *Q*-test *p*-value of 0.718.

Begg's funnel plots were generated to visually assess the potential for publication bias (Supplementary Figure 3c). Both Begg's test (*p* = 0.249) and Egger's test (*p* = 0.331) indicated no significant evidence of publication bias.

### 3.6 Association between high SII and overall survival

**Figure 7:**
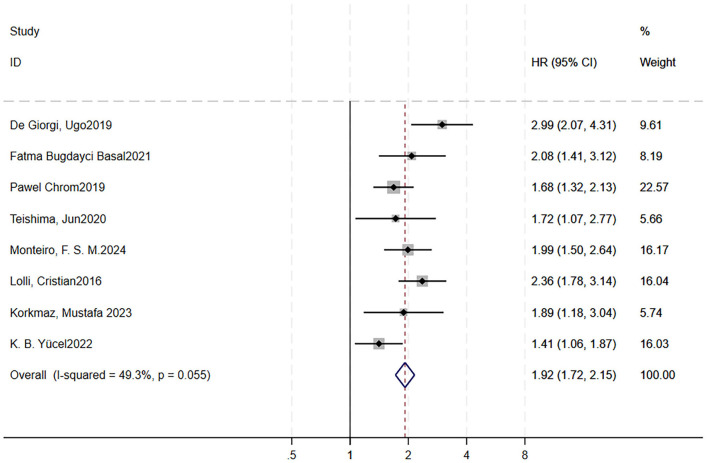
High pretreatment SII is associated with worse OS in RCC patients receiving targeted therapy. Forest plot showing the pooled HR for the association between elevated baseline SII and OS. Each study is represented by a square (point estimate) and horizontal line (95% CI). Square size corresponds to study weight in the meta-analysis. The diamond represents the pooled HR and 95% CI. The vertical dashed line indicates the null effect (HR = 1). Pooled HR = 1.92, 95% CI = 1.72–2.15, *p* < 0.001. Heterogeneity: *I*^2^ = 49.3%, *p* = 0.055. OS, overall survival; RCC, renal cell carcinoma; HR, hazard ratio; CI, confidence interval; SII, systemic immune-inflammation index.

A total of 8 studies were included to analyze the impact of high SII on OS (16, 70, 72–77). We did not analyze it for PFS, as only three articles involved it. Forest plots showed RCC patients treated with targeted therapy with high SII had shorter OS (HR = 1.92, 95% CI = 1.72–2.15, *p* < 0.0001; moderate heterogeneity, *I*^2^ = 49.3%, *p* = 0.055; Figure 7). Sensitivity analysis using the leave-one-out method showed that the pooling effect size of OS would not significantly change due to variations of a single study (Supplementary Figure 4a). A Galbraith plot indicated that two studies could be potential sources of heterogeneity (Supplementary Figure 4b). After excluding them, the forest plot showed *I*^2^ = 0%, with a *Q*-test *p*-value of 0.605.

Begg's funnel plots were generated to visually assess publication bias (Supplementary Figure 4c). Both Begg's test (*p* = 1.000) and Egger's test (*p* = 0.531) indicated no significant evidence of publication bias.

### 3.7 Association between other indicators and clinical outcomes

Beyond NLR, SII, NEU, and Hb, we also evaluated the prognostic value of other inflammatory or nutritional indicators, including CRP, PLR, BMI, Alb, PNI and the results are shown in Table 1 for OS and Table 2 for PFS.

**Table 1:** Meta-analysis results of other prognostic indicators for OS in RCC patients treated with targeted therapy.

**Indicator**	**No. of studies**	**Pooled HR (95% CI)**	***p*-Value**	***I*^2^ (%)**	***p* for heterogeneity**	**Publication bias (Egger's test, *p*)**	**Main conclusion**
CRP	21	2.34 (1.92–2.85)	<0.001	69.8	<0.001	<0.001	Significant, moderate heterogeneity and publication bias
PLR	10	1.85 (1.33–2.58)	<0.001	68.9	0.001	<0.001	Significant, moderate heterogeneity and publication bias
BMI	14	0.69 (0.59–0.79)	<0.001	52.3	0.011	0.147	Significant, moderate heterogeneity and low publication bias
Alb	8	1.53 (1.11–2.11)	0.010	68.7	0.002	0.944	Significant, moderate heterogeneity and low publication bias
PNI	6	2.00 (1.61–2.49)	<0.001	0.0	0.824	0.025	Significant, no heterogeneity and high publication bias

CRP, C-reactive protein; PLR, platelet-to-lymphocyte ratio; BMI, body mass index; Alb, albumin; PNI, prognostic nutritional index.

**Table 2:** Meta-analysis results of other prognostic indicators for PFS in RCC patients treated with targeted therapy.

**Indicator**	**No. of studies**	**Pooled HR (95% CI)**	***p*-Value**	***I*^2^ (%)**	***p* for heterogeneity**	**Publication bias (Egger's test, *p*)**	**Main conclusion**
CRP	9	1.97 (1.41–2.74)	<0.001	80	<0.001	0.003	Significant, high heterogeneity and publication bias
PLR	7	2.31 (1.28–4.18)	<0.001	86.9	<0.001	0.018	Significant, high heterogeneity and publication bias
BMI	9	0.70 (0.58–0.85)	<0.001	64.4	0.004	0.215	Significant, moderate heterogeneity and low publication bias

CRP, C-reactive protein; PLR, platelet-to-lymphocyte ratio; BMI, body mass index.

## 4 Discussion

While the prognostic value of individual inflammatory and nutritional markers in RCC has been explored in prior research, this systematic review and meta-analysis provides the first comprehensive, head-to-head comparison of nine key indicators (NLR, CRP, PLR, BMI, Alb, PNI, SII, NEU, Hb) specifically within the homogeneous context of targeted therapy. Our analysis of 152 retrospective cohort studies (*n* = 33,842) not only confirms the prognostic significance of these markers but crucially establishes an evidence-based hierarchy by evaluating both their pooled effect sizes and the consistency/robustness of the underlying data. The key findings are: (1) High NLR, high NEU, low Hb, and high SII demonstrate significant associations with poorer survival outcomes, supported by high statistical significance, low heterogeneity, and minimal publication bias, identifying them as the most robust and consistent predictors; (2) While high CRP, high PLR, low BMI, low Alb, and low PNI also show significant prognostic value, their evidence is currently limited by moderate-to-high heterogeneity or publication bias, highlighting areas where standardization and further validation are needed. This comparative framework moves beyond validating individual markers to offering practical guidance for selecting the most reliable indicators in clinical practice.

Systemic inflammation represents a prominent host-tumor interaction in cancer patients and has been shown to play a crucial role in cancer initiation, progression, metastasis, and treatment resistance (78). Numerous studies have shown that a higher inflammatory burden in cancer patients is often associated with a poorer prognosis (79, 80). For example, neutrophils, while exerting their cytotoxic function in the tumor microenvironment, also promote angiogenesis and interact with adaptive immune cells, helping tumor cells evade immune surveillance, which actually promotes tumor progression (81). Also, the high systemic SII integrating the counts of neutrophils, platelets and lymphocytes, may provide a more comprehensive depiction of this malignant network of inflammation and immunosuppression (82). Therefore, increasing baseline levels of inflammatory cells such as neutrophils, lymphocytes, and macrophages, as well as inflammatory cytokines like Interleukin-6 (IL-6) can reflect a high inflammatory state in RCC patients, thus resulting in worse survival outcomes and therapeutic effects.

The prognostic significance of nutritional indicators is closely linked to the body's energy reserves, treatment tolerance, and immune function. Malnutrition not only leads to a decline in quality of life, but is also associated with various clinical adverse consequences, such as weakened therapeutic response, increased risk of treatment toxicity, and decreased patient survival rates (83). Serum albumin is one of the most commonly used indicators of nutritional status. A low level of serum albumin indicates a persistent systemic response leading to the depletion of this protein (84). And its potential prognostic advantage lies in its low cost, repeatability, and significant effect (85). Moreover, research indicates that an increase in hemoglobin during the treatment process is associated with a poorer OS (86), while a low pretreatment Hb level can also lead to a worse prognosis (14). Furthermore, patients with RCC and a high BMI have substantial energy reserves in their adipose tissue, thereby better withstanding adverse reactions caused by targeted therapy and prolonging their survival period (87).

In line with most published meta-analyses, we found that a high baseline NLR, CRP, PLR, SII, NEU and a low pretreatment BMI, Alb, PNI, Hb were connected with shorter median OS and/or PFS with obvious statistical significance as independent indicators (10–16). However, the study conducted by Ishihara et al. (32) showed NLR was not an independent predictor of PFS, which may be because it only included second-line targeted treatments for cases where the first-line treatments failed, and the inflammatory mechanism is more complex (32). In addition, our analysis of CRP, PLR, BMI, Alb revealed moderate to high heterogeneity and CRP, PLR, PNI showed significant publication bias for OS and/or PFS, which may be due to too small sample sizes or a strong tendency to publish positive results. For those markers with larger sample sizes (NLR, NEU, Hb, SII), we use Galbraith plot to exclude studies that were identified as potential sources of heterogeneity and then their heterogeneity was well-controlled, with combined HRs being stable. We also conducted subgroup analysis to find the origin of heterogeneity but failed in cutoff value, types of targeted treatment, and histology.

It is clear to see that the SII showed the highest combined risk ratio (HR = 1.92), while the risk ratios for NEU, NLR, and Hb were slightly lower (HR range 1.74–1.88), which showed SII may be the strongest predictor of worse OS in RCC patients treated with targeted therapy. However, the differences in predictive strength among them showed no statistically significant difference (heterogeneity test between groups *p* > 0.05). Therefore, when selecting prognostic indicators in clinical practice, the absolute numerical difference in predictive strength may not be the primary consideration. Considering low heterogeneity and publication bias, the above four indicators had great predictive stability, so they may form the basis for optimal combinations. Our study is the most comprehensive meta-analysis to date that systematically assesses and compares a series of systemic inflammatory and nutritional indicators for RCC patients receiving targeted therapy, which provides the latest evidence in this field. However, the majority of the included evidence originated from retrospective observational studies. Although the quality assessment scores were generally high, the retrospective design itself limits the ability to avoid selection bias and the influence of unmeasured confounding factors, which may weaken the strength of causal inferences. In addition, this study focused on classic indicators that have accumulated a considerable amount of evidence. Some emerging prognostic markers in recent years, such as “Combination of C-Reactive Protein and Lactate Dehydrogenase with Age and Neutrophils (C-PLAN)” (88) and “Gamma-Glutamyl Transferase to Lymphocyte Ratio (GLR)” (89) and so on, although showing potential in preliminary retrospective studies, were not quantitatively synthesized in this meta-analysis due to the limited number of relevant studies and small sample sizes. As more evidence accumulates in the future, these indicators are expected to become promising emerging predictive tools.

## 5 Conclusion

NLR, NEU, SII and Hb are robust and consistent prognostic predictors for patients with RCC who receive targeted therapy. Although CRP, PLR, BMI, Alb, and PNI also demonstrate prognostic value, there are issues of heterogeneity and publication bias when integrating their evidence. The evidence included in this study all originated from retrospective observational studies, and thus selection bias and the influence of unmeasured confounding factors could not be fully avoided. These findings provide evidence-based guidance for selecting risk stratification tools in clinical practice and are helpful for improving treatment decisions and prognosis management for patients with RCC.

## Data Availability

The original contributions presented in the study are included in the article/Supplementary material, further inquiries can be directed to the corresponding authors.
