# Supplementary material for: The prognostic impact of systemic inflammation and nutritional indicators on targeted therapy for renal cell carcinoma: a systematic review and meta-analysis
Source: Front Nutr. 2026 Feb 25;13:1777753. doi: 10.3389/fnut.2026.1777753 (PMC12975561; doi:10.3389/fnut.2026.1777753)
Supplement: Supplementary file 2 [file Table_2.docx]

| **Supplemental Table 2** Search Strategies in Six Databases | |
| --- | --- |
| Database | Search strategies |
| PubMed | ("Carcinoma, Renal Cell"[MeSH] OR "Kidney Neoplasms"[MeSH] OR "Renal Cell Carcinoma"[TIAB] OR "Kidney Cancer"[TIAB] OR "renal cancer"[TIAB] OR "renal adenocarcinoma"[TIAB] OR "clear cell renal cell carcinoma"[TIAB] OR ccRCC[TIAB] OR RCC[TIAB])  **AND** ("Inflammation"[MeSH] OR "Inflammation Mediators"[MeSH] OR "Systemic Inflammatory Response Syndrome"[MeSH] OR "Nutrition Assessment"[MeSH] OR "Albumins"[MeSH] OR "Body Mass Index"[MeSH] OR "BMI"[TIAB] OR "Prognostic Nutritional Index"[TIAB] OR PNI[TIAB] OR "Neutrophil to Lymphocyte Ratio"[TIAB] OR NLR[TIAB] OR "Platelet to Lymphocyte Ratio"[TIAB] OR PLR[TIAB] OR "Systemic Immune Inflammation Index"[TIAB] OR SII[TIAB] OR "Lymphocyte to Monocyte Ratio"[TIAB] OR LMR[TIAB] OR "C-reactive protein"[TIAB] OR CRP[TIAB] OR "C-reactive protein-albumin ratio"[TIAB] OR "CRP to albumin ratio"[TIAB] OR CAR[TIAB] OR "glasgow prognostic score"[TIAB] OR "modified glasgow prognostic score"[TIAB] OR GPS[TIAB] OR mGPS[TIAB] OR "Controlling Nutritional Status"[TIAB] OR CONUT[TIAB] OR "Advanced Lung Cancer Inflammation Index"[TIAB] OR ALI[TIAB])  **AND** ("Molecular Targeted Therapy"[MeSH] OR "Tyrosine Kinase Inhibitors"[MeSH] OR "mTOR Inhibitors"[MeSH] OR "Vascular Endothelial Growth Factor A"[MeSH] OR "Targeted Therapy"[TIAB] OR TKI[TIAB] OR "VEGF Inhibitors"[TIAB] OR Sunitinib[TIAB] OR Pazopanib[TIAB] OR Axitinib[TIAB] OR Sorafenib[TIAB] OR Everolimus[TIAB] OR Temsirolimus[TIAB] OR Lenvatinib[TIAB] OR Cabozantinib[TIAB] OR Bevacizumab[TIAB]) |
| EMBASE | #1. ("Carcinoma, Renal Cell" or "Kidney Neoplasms" or "Renal Cell Carcinoma" or "Kidney Cancer" or "renal cancer" or "renal adenocarcinoma" or "clear cell renal cell carcinoma" or ccRCC or RCC).af.  #2. ("Inflammation" or "Inflammation Mediators" or "Systemic Inflammatory Response Syndrome" or "Nutrition Assessment" or "Albumins" or "Body Mass Index" or "BMI" or "Prognostic Nutritional Index" or PNI or "Neutrophil to Lymphocyte Ratio" or NLR or "Platelet to Lymphocyte Ratio" or PLR or "Systemic Immune Inflammation Index" or SII or "Lymphocyte to Monocyte Ratio" or LMR or "C-reactive protein" or CRP or "C-reactive protein-albumin ratio" or "CRP to albumin ratio" or CAR or "glasgow prognostic score" or "modified glasgow prognostic score" or GPS or mGPS or "Controlling Nutritional Status" or CONUT or "Advanced Lung Cancer Inflammation Index" or ALI).af.  #3. ("Molecular Targeted Therapy" or "Tyrosine Kinase Inhibitors" or "mTOR Inhibitors" or "Vascular Endothelial Growth Factor A" or "Targeted Therapy" or TKI or "VEGF Inhibitors" or Sunitinib or Pazopanib or Axitinib or Sorafenib or Everolimus or Temsirolimus or Lenvatinib or Cabozantinib or Bevacizumab).af.  #4. #1 AND #2 AND #3 |
| Cochrane Library | #1 ("Carcinoma, Renal Cell").af  #2 ("Kidney Neoplasms").af  #3 ("Renal Cell Carcinoma").af  #4 ("Kidney Cancer").af  #5 ("renal cancer").af  #6 ("renal adenocarcinoma").af  #7 ("clear cell renal cell carcinoma").af  #8 (ccRCC).af  #9 (RCC).af  #10 #1 OR #2 OR #3 OR #4 OR #5 OR #6 OR #7 OR #8 OR #9  #11 ("Inflammation").af  #12 ("Inflammation Mediators").af  #13 ("Systemic Inflammatory Response Syndrome").af  #14 ("Nutrition Assessment").af  #15 ("Albumins").af  #16 ("Body Mass Index").af  #17 (BMI).af  #18 ("Prognostic Nutritional Index").af  #19 (PNI).af  #20 ("Neutrophil to Lymphocyte Ratio").af  #21 (NLR).af  #22 ("Platelet to Lymphocyte Ratio").af  #23 (PLR).af  #24 ("Systemic Immune Inflammation Index").af  #25 (SII).af  #26 ("Lymphocyte to Monocyte Ratio").af  #27 (LMR).af  #28 ("C-reactive protein").af  #29 (CRP).af  #30 ("C-reactive protein-albumin ratio").af  #31 ("CRP to albumin ratio").af  #32 (CAR).af  #33 ("glasgow prognostic score").af  #34 (GPS).af  #35 ("modified glasgow prognostic score").af  #36 (mGPS).af  #37 ("Controlling Nutritional Status").af  #38 (CONUT).af  #39 ("Advanced Lung Cancer Inflammation Index").af  #40 (ALI).af  #41 #11 OR #12 OR #13 OR #14 OR #15 OR #16 OR #17 OR #18 OR #19 OR #20 OR #21 OR #22 OR #23 OR #24 OR #25 OR #26 OR #27 OR #28 OR #29 OR #30 OR #31 OR #32 OR #33 OR #34 OR #35 OR #36 OR #37 OR #38 OR #39 OR #40  #42 ("Molecular Targeted Therapy").af  #43 ("Tyrosine Kinase Inhibitors").af  #44 ("mTOR Inhibitors").af  #45 ("Vascular Endothelial Growth Factor A").af  #46 ("Targeted Therapy").af  #47 (TKI).af  #48 ("VEGF Inhibitors").af  #49 (Sunitinib).af  #50 (Pazopanib).af  #51 (Axitinib).af  #52 (Sorafenib).af  #53 (Everolimus).af  #54 (Temsirolimus).af  #55 (Lenvatinib).af  #56 (Cabozantinib).af  #57 (Bevacizumab).af  #58 #42 OR #43 OR #44 OR #45 OR #46 OR #47 OR #48 OR #49 OR #50 OR #51 OR #52 OR #53 OR #54 OR #55 OR #56 OR #57  #59 #10 AND #41 AND #58 |
| Web of Science Core Collection | #1 ("Carcinoma, Renal Cell" or "Kidney Neoplasms" or "Renal Cell Carcinoma" or "Kidney Cancer" or "renal cancer" or "renal adenocarcinoma" or "clear cell renal cell carcinoma" or ccRCC or RCC) (All Fields)  #2 ("Inflammation" or "Inflammation Mediators" or "Systemic Inflammatory Response Syndrome" or "Nutrition Assessment" or "Albumins" or "Body Mass Index" or "BMI" or "Prognostic Nutritional Index" or PNI or "Neutrophil to Lymphocyte Ratio" or NLR or "Platelet to Lymphocyte Ratio" or PLR or "Systemic Immune Inflammation Index" or SII or "Lymphocyte to Monocyte Ratio" or LMR or "C-reactive protein" or CRP or "C-reactive protein-albumin ratio" or "CRP to albumin ratio" or CAR or "glasgow prognostic score" or "modified glasgow prognostic score" or GPS or maps or "Controlling Nutritional Status" or CONUT or "Advanced Lung Cancer Inflammation Index" or ALI) (All Fields)  #3 ("Molecular Targeted Therapy" or "Tyrosine Kinase Inhibitors" or "mTOR Inhibitors" or "Vascular Endothelial Growth Factor A" or "Targeted Therapy" or TKI or "VEGF Inhibitors" or Sunitinib or Pazopanib or Axitinib or Sorafenib or Everolimus or Temsirolimus or Lenvatinib or Cabozantinib or Bevacizumab) (All Fields)  #4 #1 AND #2 AND #3 |
| Scopus | ("Carcinoma, Renal Cell" or "Kidney Neoplasms" or "Renal Cell Carcinoma" or "Kidney Cancer" or "renal cancer" or "renal adenocarcinoma" or "clear cell renal cell carcinoma" or ccRCC or RCC) (All Fields)  and ("Inflammation" or "Inflammation Mediators" or "Systemic Inflammatory Response Syndrome" or "Nutrition Assessment" or "Albumins" or "Body Mass Index" or "BMI" or "Prognostic Nutritional Index" or PNI or "Neutrophil to Lymphocyte Ratio" or NLR or "Platelet to Lymphocyte Ratio" or PLR or "Systemic Immune Inflammation Index" or SII or "Lymphocyte to Monocyte Ratio" or LMR or "C-reactive protein" or CRP or "C-reactive protein-albumin ratio" or "CRP to albumin ratio" or CAR or "glasgow prognostic score" or "modified glasgow prognostic score" or GPS or maps or "Controlling Nutritional Status" or CONUT or "Advanced Lung Cancer Inflammation Index" or ALI) (All Fields)  and ("Molecular Targeted Therapy" or "Tyrosine Kinase Inhibitors" or "mTOR Inhibitors" or "Vascular Endothelial Growth Factor A" or "Targeted Therapy" or TKI or "VEGF Inhibitors" or Sunitinib or Pazopanib or Axitinib or Sorafenib or Everolimus or Temsirolimus or Lenvatinib or Cabozantinib or Bevacizumab) (All Fields) |
| Medline | ("Carcinoma, Renal Cell" or "Kidney Neoplasms" or "Renal Cell Carcinoma" or "Kidney Cancer" or "renal cancer" or "renal adenocarcinoma" or "clear cell renal cell carcinoma" or ccRCC or RCC) (All Fields)  and ("Inflammation" or "Inflammation Mediators" or "Systemic Inflammatory Response Syndrome" or "Nutrition Assessment" or "Albumins" or "Body Mass Index" or "BMI" or "Prognostic Nutritional Index" or PNI or "Neutrophil to Lymphocyte Ratio" or NLR or "Platelet to Lymphocyte Ratio" or PLR or "Systemic Immune Inflammation Index" or SII or "Lymphocyte to Monocyte Ratio" or LMR or "C-reactive protein" or CRP or "C-reactive protein-albumin ratio" or "CRP to albumin ratio" or CAR or "glasgow prognostic score" or "modified glasgow prognostic score" or GPS or maps or "Controlling Nutritional Status" or CONUT or "Advanced Lung Cancer Inflammation Index" or ALI) (All Fields)  and ("Molecular Targeted Therapy" or "Tyrosine Kinase Inhibitors" or "mTOR Inhibitors" or "Vascular Endothelial Growth Factor A" or "Targeted Therapy" or TKI or "VEGF Inhibitors" or Sunitinib or Pazopanib or Axitinib or Sorafenib or Everolimus or Temsirolimus or Lenvatinib or Cabozantinib or Bevacizumab) (All Fields) |
