# Supplementary material for: The prognostic impact of systemic inflammation and nutritional indicators on targeted therapy for renal cell carcinoma: a systematic review and meta-analysis
Source: Front Nutr. 2026 Feb 25;13:1777753. doi: 10.3389/fnut.2026.1777753 (PMC12975561; doi:10.3389/fnut.2026.1777753)
Supplement: Supplementary file 5 [file Table_5.docx]

Table Newcastle-Ottawa Quality Assessments Scale

| Study | Quality indicators from Newcastle-Ottawa Scale | | | | | | | | Scores |
| --- | --- | --- | --- | --- | --- | --- | --- | --- | --- |
|  | 1 | 2 | 3 | 4 | 5 | 6 | 7 | 8 |  |
| Kosuke Ueda 2022 | * | * | * | - | ** | * | * | * | 8 |
| MAKI YOSHINO 2021 | * | * | * | - | ** | * | * | * | 8 |
| Ryuichi Mizuno 2017 | * | * | * | - | ** | * | * | * | 8 |
| Toshiki Ito 2022 | * | * | * | - | ** | * | * | * | 8 |
| Kohei Takei 2023 | * | * | * | - | ** | * | * | * | 8 |
| Mehmet Asim Bilen 2018 | * | * | * | - | * | * | * | * | 7 |
| Ahmet Yildirim 2025 | * | * | * | - | ** | * | * | * | 8 |
| Matthew D Tucker 2021 | * | * | * | - | ** | * | * | * | 8 |
| Rohit Vivek Goswamy 2024 | * | * | * | - | ** | * | * | * | 8 |
| Jun Wang 2023 | * | * | * | - | ** | * | * | * | 8 |
| Yoshihiko Tomita 2021 | * | * | * | - | - | * | * | * | 6 |
| Dylan J Martini 2019 | * | * | * | - | * | * | * | * | 7 |
| Jacqueline T Brown 2020 | * | * | * | - | ** | * | * | * | 8 |
| Marc Eid 2023 | * | * | * | - | * | * | * | * | 7 |
| Mehmet Asim Bilen 2022 | * | * | * | - | - | * | * | * | 7 |
| Wen Cai 2017 | * | * | * | - | ** | * | * | * | 8 |
| Daniel Keizman 2012 | * | * | * | - | ** | * | * | * | 8 |
| De Giorgi, Ugo 2019 | * | * | * | - | ** | * | * | * | 8 |
| Yasser Ged 2022 | * | * | * | - | ** | * | * | * | 8 |
| Brian W Labadie 2019 | * | * | * | - | ** | * | * | * | 8 |
| Dylan J Martini 2021 | * | * | * | - | ** | * | * | * | 8 |
| Yan Song 2016 | * | * | * | - | ** | * | * | * | 8 |
| Laurence Albiges 2016 | * | - | * | - | ** | * | * | * | 7 |
| Nicholas M Donin 2016 | * | * | * | - | ** | * | * | * | 8 |
| Peter J. Goebell M.D., P.D. 2018 | * | * | * | - | ** | * | * | * | 8 |
| Matteo Santoni 2021 | * | * | * | - | ** | * | * | * | 8 |
| Scott Thomas Colville Shepherd 2017 | * | * | * | - | ** | * | * | * | 8 |
| Fatma Bugdayci Basal 2021 | * | * | * | - | ** | * | * | * | 8 |
| Arnab Basu 2021 | * | * | * | - | ** | * | * | * | 8 |
| Minoru Kobayashi 2013 | * | * | * | - | * | * | * | * | 7 |
| Kimiharu Takamatsu 2018 | * | * | * | - | ** | * | * | * | 8 |
| Shingo Toyoda 2024 | * | * | * | - | ** | * | * | * | 8 |
| Bradley J Atkinson 2014 | * | * | * | - | ** | * | * | * | 8 |
| Matteo Santoni 2023 | * | * | * | - | ** | * | * | * | 8 |
| Francesco Pierantoni 2021 | * | * | * | - | ** | * | * | * | 8 |
| Kojiro Ohba 2023 | * | * | * | - | ** | * | * | * | 8 |
| E. Lanoy 2014 | * | * | * | - | ** | * | * | * | 8 |
| Elisa Kankkunen 2022 | * | * | * | - | ** | * | * | * | 8 |
| Eduard Roussel 2021 | * | * | * | - | ** | * | * | * | 8 |
| Wen-Hao Xu 2019 | * | * | * | - | ** | * | * | * | 8 |
| Sakae Konishi 2019 | * | * | * | - | ** | * | * | * | 8 |
| Sung Han Kim 2018 | * | * | * | - | ** | * | * | * | 8 |
| Sandra Steffens 2011 | * | * | * | - | * | * | * | * | 7 |
| Mustafa Korkmaz 2023 | * | * | * | - | ** | * | * | * | 8 |
| Weijie Gu 2017 | * | * | * | - | ** | * | * | * | 8 |
| Hideaki Miyake 2016 | * | * | * | - | ** | * | * | * | 8 |
| Jun Teishima 2017 | * | * | * | - | ** | * | * | * | 8 |
| Hiroki Ishihara 2017 | * | * | * | - | ** | * | * | * | 8 |
| Nahjatul Kursyiah Abd Ghafar 2018 | * | * | * | - | ** | * | * | * | 8 |
| R. Mizuno 2023 | * | * | * | - | ** | * | * | * | 8 |
| Hong-Kai Wang 2016 | * | * | * | - | ** | * | * | * | 8 |
| Hai-Liang Zhang 2011 | * | * | * | - | - | * | * | * | 6 |
| Hatice Bolek 2025 | * | * | * | - | * | * | * | * | 7 |
| Yasuyoshi Okamura 2019 | * | * | * | - | ** | * | * | * | 8 |
| Keita Tamura 2021 | * | * | * | - | ** | * | * | * | 8 |
| Pawel Chrom 2019 | * | * | * | - | ** | * | * | * | 8 |
| Wang, Hongkai 2022 | * | - | * | - | ** | * | * | * | 7 |
| Tamura, Keita 2020 | * | * | * | - | ** | * | * | * | 8 |
| Chrom, P. 2016 | * | * | * | - | ** | * | * | * | 8 |
| Watari, S. 2023 | * | * | * | - | ** | * | * | * | 8 |
| Aktepe, O. H. 2021 | * | * | * | - | ** | * | * | * | 8 |
| Mizuno, Ryuichi 2017 | * | * | * | - | ** | * | * | * | 8 |
| Ishihara, Hiroki 2024 | * | * | * | - | ** | * | * | * | 8 |
| Ishihara, H. 2022 | * | * | * | - | - | * | * | * | 6 |
| Teishima, Jun 2016 | * | * | * | - | ** | * | * | * | 8 |
| Saito, Kazutaka 2009 | * | * | * | - | ** | * | * | * | 8 |
| Nakane, Keita 2025 | * | * | * | - | ** | * | * | * | 8 |
| Ito, Keiichi 2019 | * | * | * | - | ** | * | * | * | 8 |
| Takemoto, Kenshiro 2023 | * | * | * | - | ** | * | * | * | 8 |
| Yukihiro, Kazuma 2022 | * | * | * | - | ** | * | * | * | 8 |
| Tjokrowidjaja, Angelina 2020 | * | * | * | - | ** | * | * | * | 8 |
| Teishima, Jun 2017 | * | * | * | - | ** | * | * | * | 8 |
| Ozaki, Keisuke 2023 | * | * | * | - | ** | * | * | * | 8 |
| Teishima, Jun 2020 | * | * | * | - | ** | * | * | * | 8 |
| Saal, Jonas 2023 | * | - | * | - | ** | * | * | * | 7 |
| Colomba, E. 2020 | * | * | * | - | - | * | * | * | 6 |
| Silva, C. A. C. 2023 | * | * | * | - | ** | * | * | * | 8 |
| Fujiwara, R. 2021 | * | * | * | - | ** | * | * | * | 8 |
| Yukihiro, Kazuma 2025 | * | * | * | - | ** | * | * | * | 8 |
| Cortellini, Alessio 2019 | * | * | * | - | ** | * | * | * | 8 |
| Jeyakumar, Ghayathri 2017 | * | * | * | - | ** | * | * | * | 8 |
| Kim, S. H. 2018 | * | * | * | - | ** | * | * | * | 8 |
| Naito, S. 2020 | * | * | * | - | ** | * | * | * | 8 |
| Young, Matthew 2024 | * | * | * | - | ** | * | * | * | 8 |
| Teishima, Jun 2018 | * | * | * | - | ** | * | * | * | 8 |
| Fujita, T. 2016 | * | * | * | - | - | * | * | * | 6 |
| Ishihara, Hiroki 2016 | * | * | * | - | * | * | * | * | 7 |
| Shirotake, S. 2019 | * | * | * | - | ** | * | * | * | 8 |
| Cai, W. 2017 | * | * | * | - | ** | * | * | * | 8 |
| Antoun, S. 2013 | * | * | * | - | ** | * | * | * | 8 |
| Monteiro, F. S. M. 2024 | * | * | * | - | ** | * | * | * | 8 |
| Lolli, Cristian 2016 | * | * | * | - | ** | * | * | * | 8 |
| Wang, Zhaojuan 2023 | * | * | * | - | ** | * | * | * | 8 |
| Korkmaz, Mustafa 2023 | * | * | * | - | ** | * | * | * | 8 |
| Harris, Wayne B. 2017 | * | * | * | - | ** | * | * | * | 8 |
| Kikuchi, Hiroshi 2025 | * | * | * | - | ** | * | * | * | 8 |
| Pérez-Valderrama, B. 2016 | * | * | * | - | ** | * | * | * | 8 |
| Ladoire, Sylvain 2011 | * | * | * | - | ** | * | * | * | 8 |
| O. H. Aktepe 2021 | * | * | * | - | ** | * | * | * | 8 |
| Oktay Halit Aktepe 2021 | * | * | * | - | ** | * | * | * | 8 |
| Edouard Auclin 2017 | * | * | * | - | ** | * | * | * | 8 |
| İbrahim Vedat Bayoğlu 2023 | * | * | * | - | ** | * | * | * | 8 |
| Benoit Beuselinck 2013 | * | * | * | - | ** | * | * | * | 8 |
| E. Bolzacchini 2022 | * | * | * | - | ** | * | * | * | 8 |
| Wen Cai 2017 | * | * | * | - | ** | * | * | * | 8 |
| B. Cetin 2012 | * | * | * | - | ** | * | * | * | 8 |
| M. Coriano 2025 | * | - | * | - | ** | * | * | * | 7 |
| T. Fujita 2017 | * | * | * | - | ** | * | * | * | 8 |
| L. Gil 2022 | * | * | * | - | ** | * | * | * | 8 |
| G. C. Giudice 2025 | * | * | * | - | ** | * | * | * | 8 |
| W. J. Gu 2015 | * | * | * | - | ** | * | * | * | 8 |
| S. Gunduz 2014 | * | * | * | - | ** | * | * | * | 8 |
| D. Y. C. Heng 2009 | * | * | * | - | ** | * | * | * | 8 |
| J. Huszno 2019 | * | * | * | - | ** | * | * | * | 8 |
| H. Ishihara 2019 | * | * | * | - | ** | * | * | * | 8 |
| T. Köşeci, M 2025 | * | * | * | - | ** | * | * | * | 8 |
| Y. Kusuda 2013 | * | * | * | - | ** | * | * | * | 8 |
| W. A. Kwon 2017 | * | * | * | - | ** | * | * | * | 8 |
| S. Lee 2024 | * | * | * | - | ** | * | * | * | 8 |
| G. N. Marta 2020 | * | * | * | - | ** | * | * | * | 8 |
| Y. Matsushita 2024 | * | * | * | - | ** | * | * | * | 8 |
| R. Motzer 2013 | * | * | * | - | ** | * | * | * | 8 |
| K. Ning 2022 | * | * | * | - | ** | * | * | * | 8 |
| A. B. Nixon 2022 | * | * | * | - | ** | * | * | * | 8 |
| K. Ohba 2024 | * | * | * | - | ** | * | * | * | 8 |
| T. J. Park 2016 | * | * | * | - | ** | * | * | * | 8 |
| A. I. Parosanu 2023 | * | * | * | - | ** | * | * | * | 8 |
| A. Qaisar 2023 | * | * | * | - | ** | * | * | * | 8 |
| S. E. Rebuzzi 2022 | * | * | * | - | ** | * | * | * | 8 |
| E. Roussel 2020 | * | * | * | - | ** | * | * | * | 8 |
| A. Sacré 2016 | * | * | * | - | ** | * | * | * | 8 |
| G. Sahin 2025 | * | * | * | - | ** | * | * | * | 8 |
| M. Santoni 2015 | * | * | * | - | ** | * | * | * | 8 |
| M. Santoni 2013 | * | * | * | - | ** | * | * | * | 8 |
| K. Sato 2025 | * | * | * | - | ** | * | * | * | 8 |
| M. T. Sato 2022 | * | * | * | - | ** | * | * | * | 8 |
| S. Y. Tacar 2022 | * | * | * | - | ** | * | * | * | 8 |
| K. Takamatsu 2018 | * | * | * | - | ** | * | * | * | 8 |
| K. Takamatsu 2019 | * | * | * | - | ** | * | * | * | 8 |
| K. Takemura 2020 | * | * | * | - | ** | * | * | * | 8 |
| N. Tanaka 2017 | * | * | * | - | ** | * | * | * | 8 |
| J. Teishima 2014 | * | * | * | - | ** | * | * | * | 8 |
| Z. Tomčová 2025 | * | * | * | - | ** | * | * | * | 8 |
| C. Tommasi 2023 | * | * | * | - | ** | * | * | * | 8 |
| A. Tripathi 2017 | * | * | * | - | ** | * | * | * | 8 |
| K. Ueda 2020 | * | * | * | - | ** | * | * | * | 8 |
| B. Wang 2019 | * | * | * | - | ** | * | * | * | 8 |
| K. Xu 2020 | * | * | * | - | ** | * | * | * | 8 |
| Y. Yasuda 2013 | * | * | * | - | ** | * | * | * | 8 |
| A. Yoshimura 2024 | * | * | * | - | ** | * | * | * | 8 |
| K. B. Yücel 2022 | * | * | * | - | ** | * | * | * | 8 |
| G. M. Zhang, 2016 | ***** | ***** | ***** | **-** | ** | ***** | ***** | ***** | 8 |

1, Representativeness of the exposed cohort; 2, Selection of the non-exposed cohort; 3, Ascertainment of exposure; 4, Demonstration that outcome of interest was not present at start of study; 5, Comparability of cohorts on the basis of the design or analysis; 6, Assessment of outcome; 7, Was follow-up long enough for outcomes to occur; 8, Adequacy of follow up of cohorts. *, each quality choice could be awarded a maximum of one star except for the numbered 5 item which could be granted a maximum of two stars. If the final score >6 stars, we regarded it as high quality
