# Supplementary material for: The prognostic impact of systemic inflammation and nutritional indicators on targeted therapy for renal cell carcinoma: a systematic review and meta-analysis
Source: Front Nutr. 2026 Feb 25;13:1777753. doi: 10.3389/fnut.2026.1777753 (PMC12975561; doi:10.3389/fnut.2026.1777753)
Supplement: Supplementary file 6 [file Image_1.pdf]

Supplementary Figures

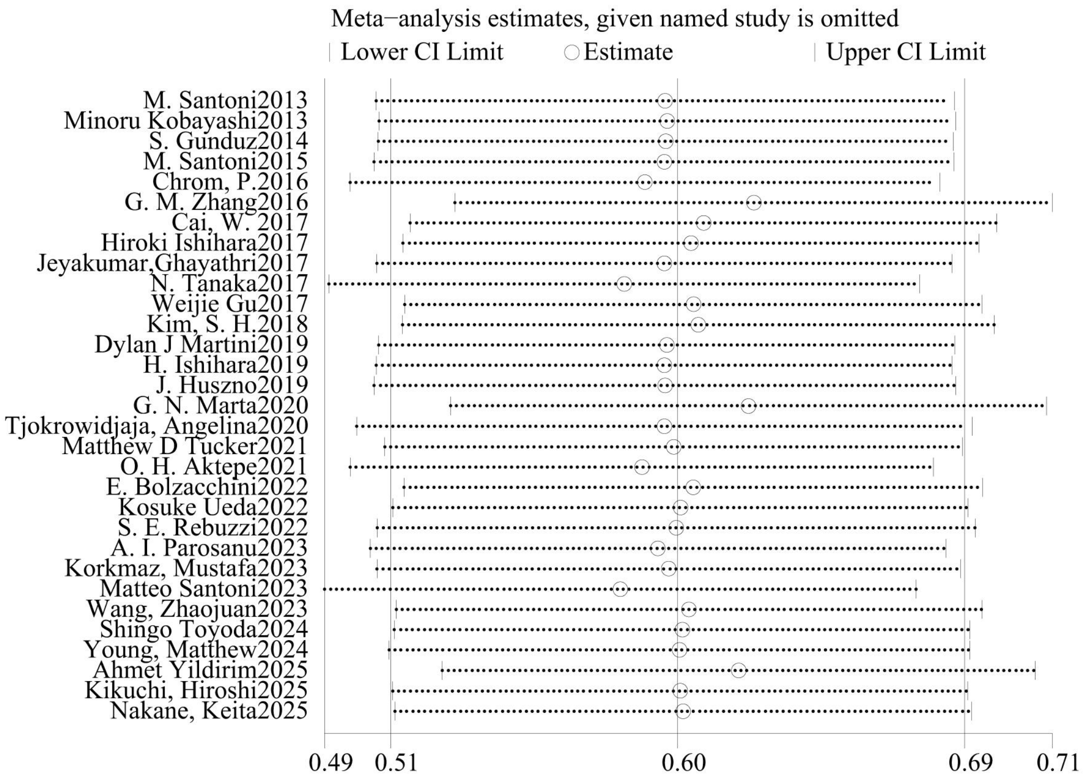

Supplementary Figure 1a. Sensitivity analysis of OS in RCC patients treated with targeted therapy with high NLR versus low NLR.

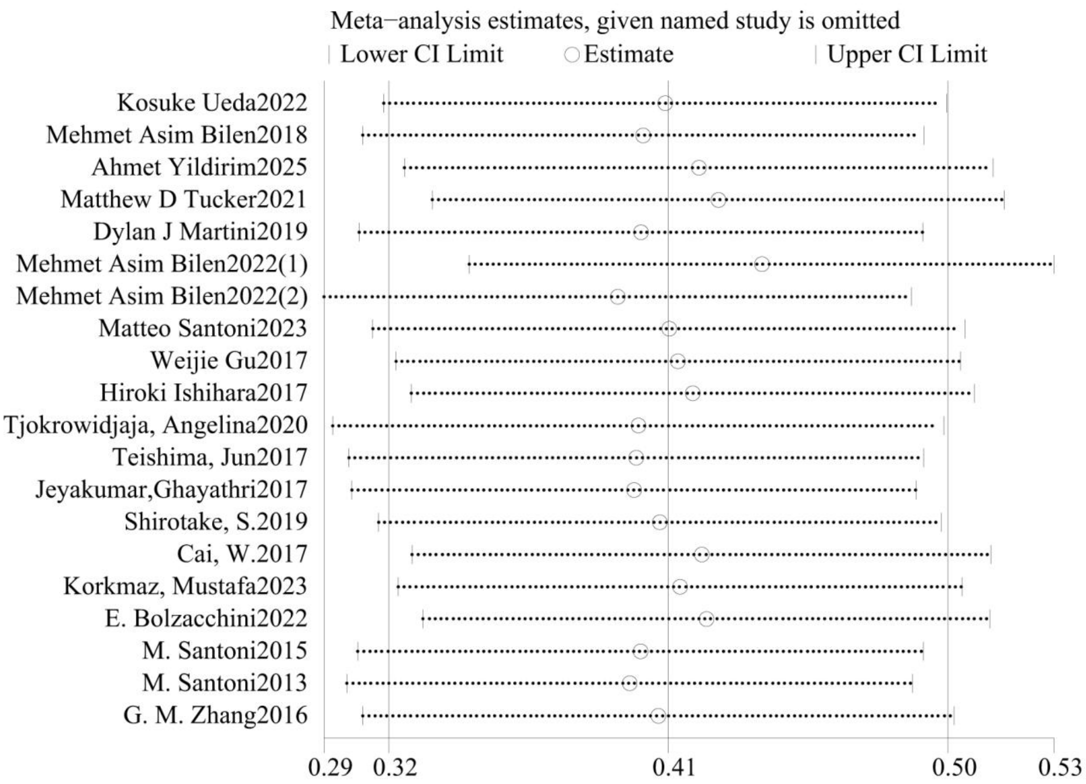

Supplementary Figure 1b. Sensitivity analysis of PFS in RCC patients treated with targeted therapy with high NLR versus low NLR.

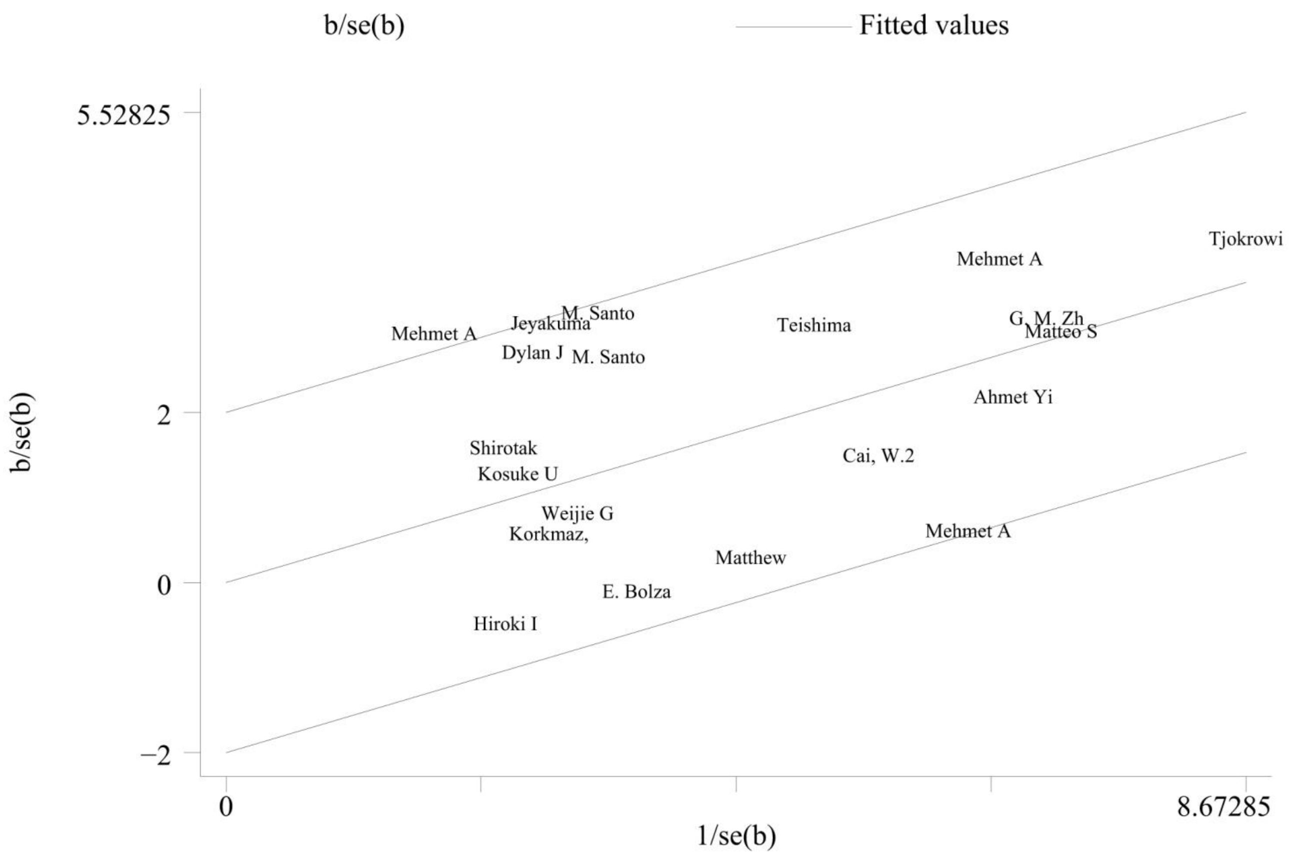

Supplementary Figure 1c. Galbraith plot of PFS in RCC patients treated with targeted therapy with high NLR versus low NLR.

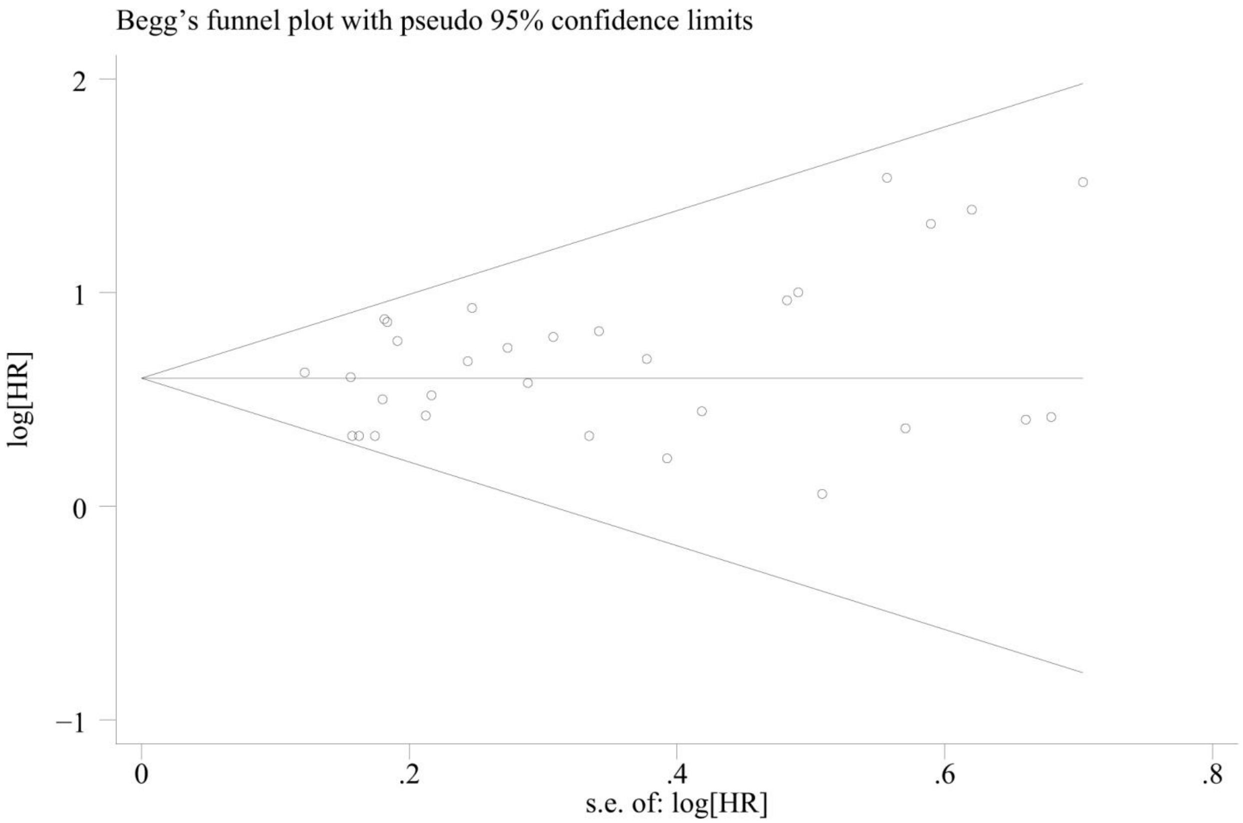

Supplementary Figure 1d. Begg's funnel plot of OS in RCC patients treated with targeted therapy with high NLR versus low NLR.

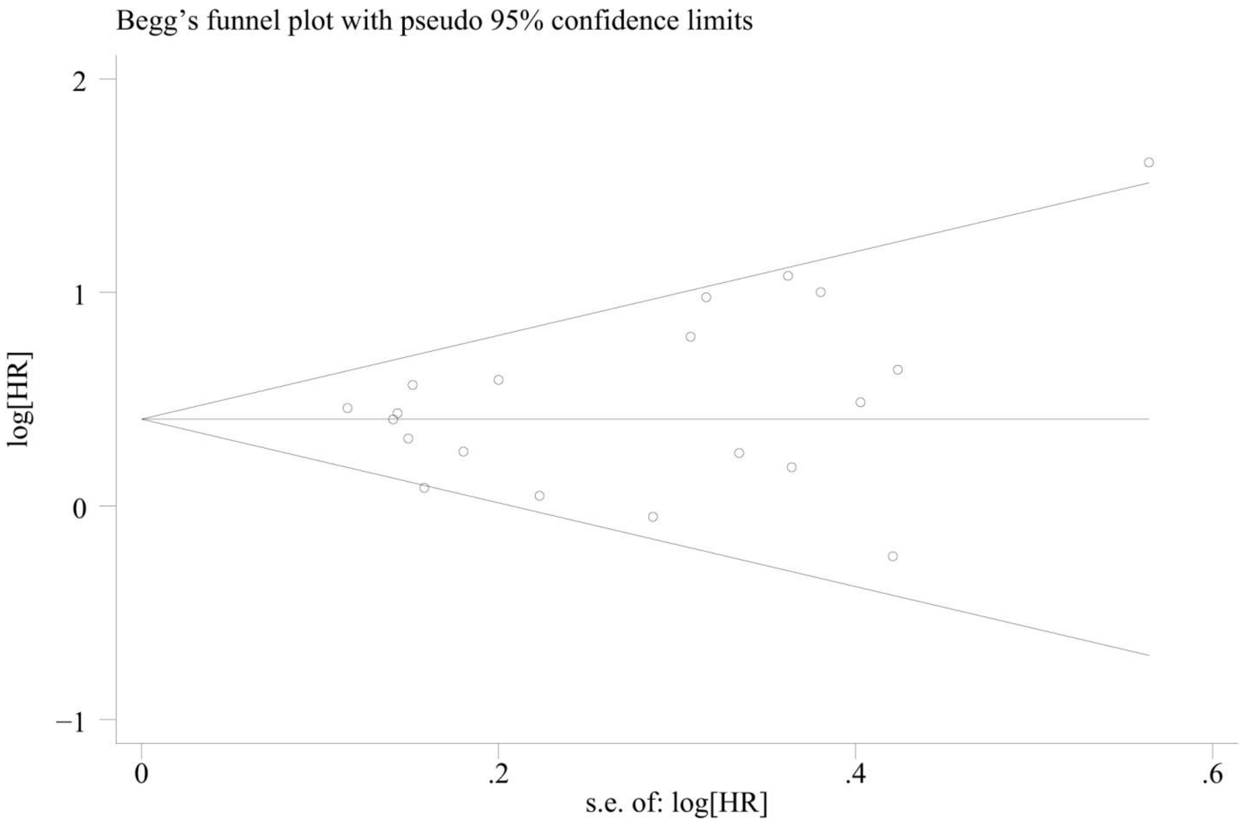

Supplementary Figure 1e. Begg's funnel plot of PFS in RCC patients treated with targeted therapy with high NLR versus low NLR.

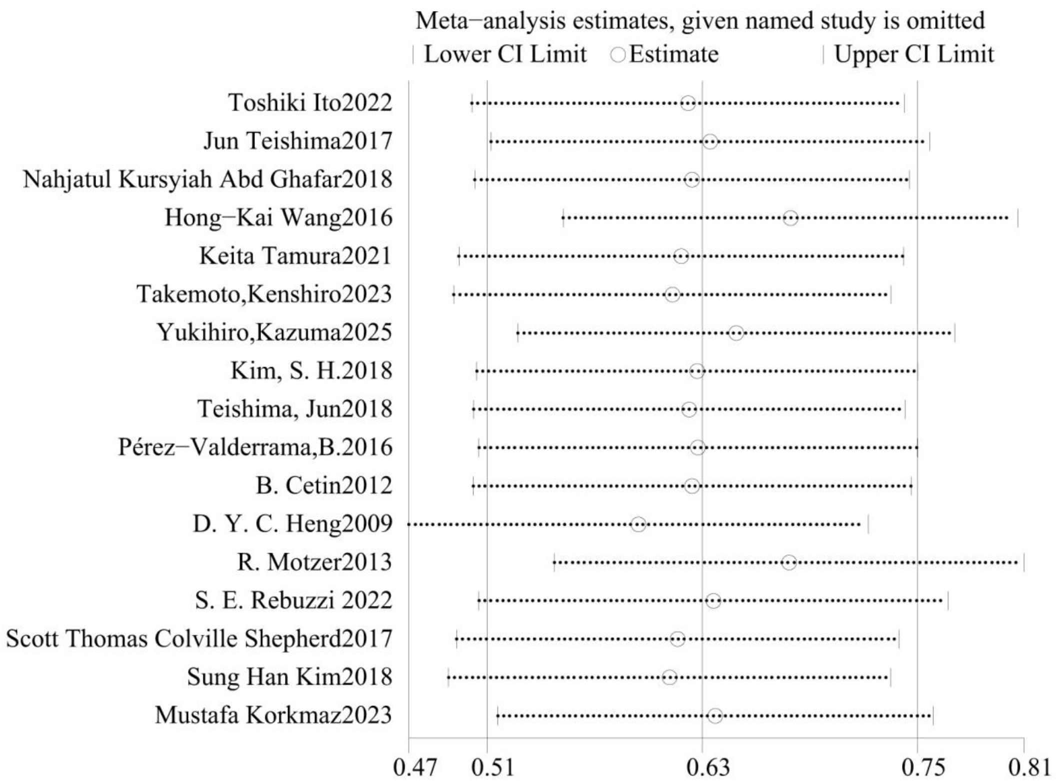

Supplementary Figure 2a. Sensitivity analysis of OS in RCC patients treated with targeted therapy with high NEU versus low NEU.

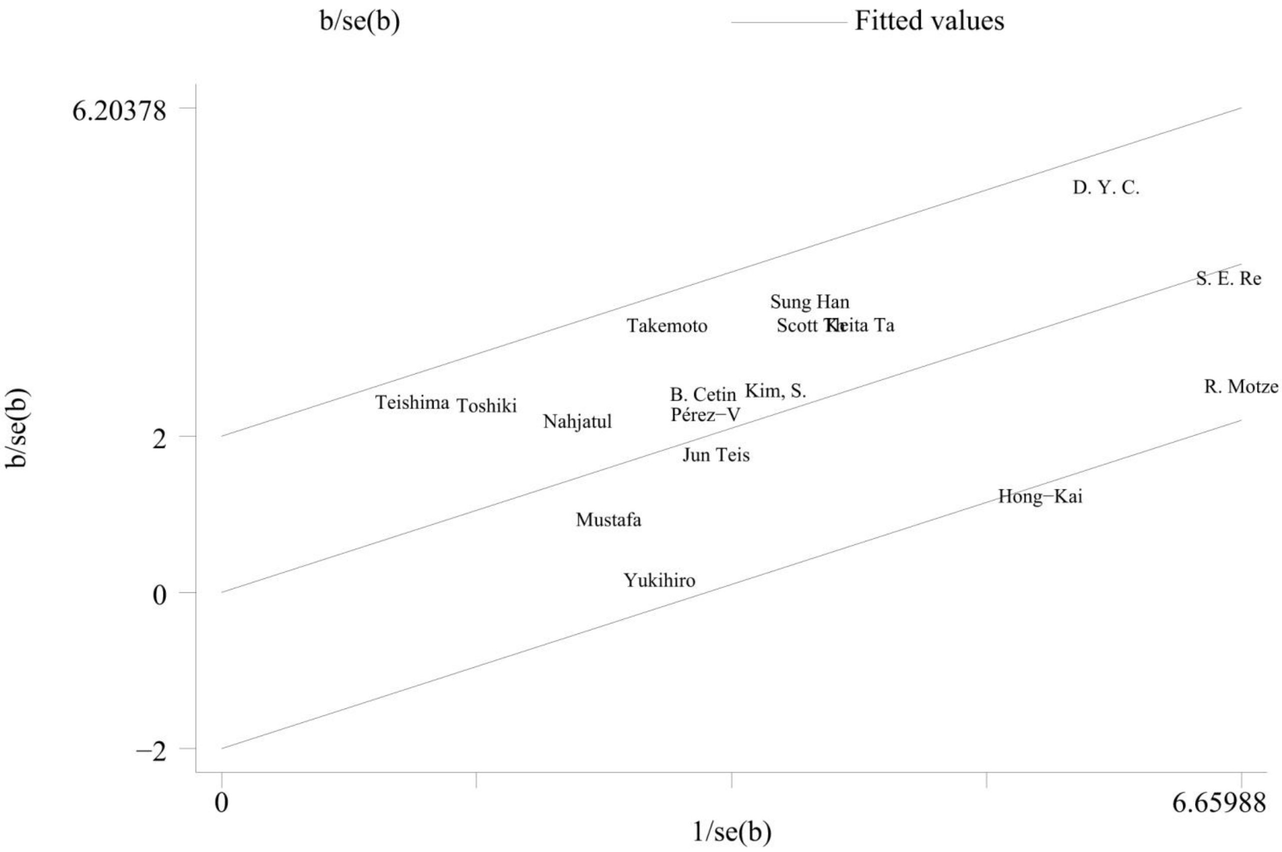

Supplementary Figure 2b. Galbraith plot of OS in RCC patients treated with targeted therapy with high NEU versus low NEU.

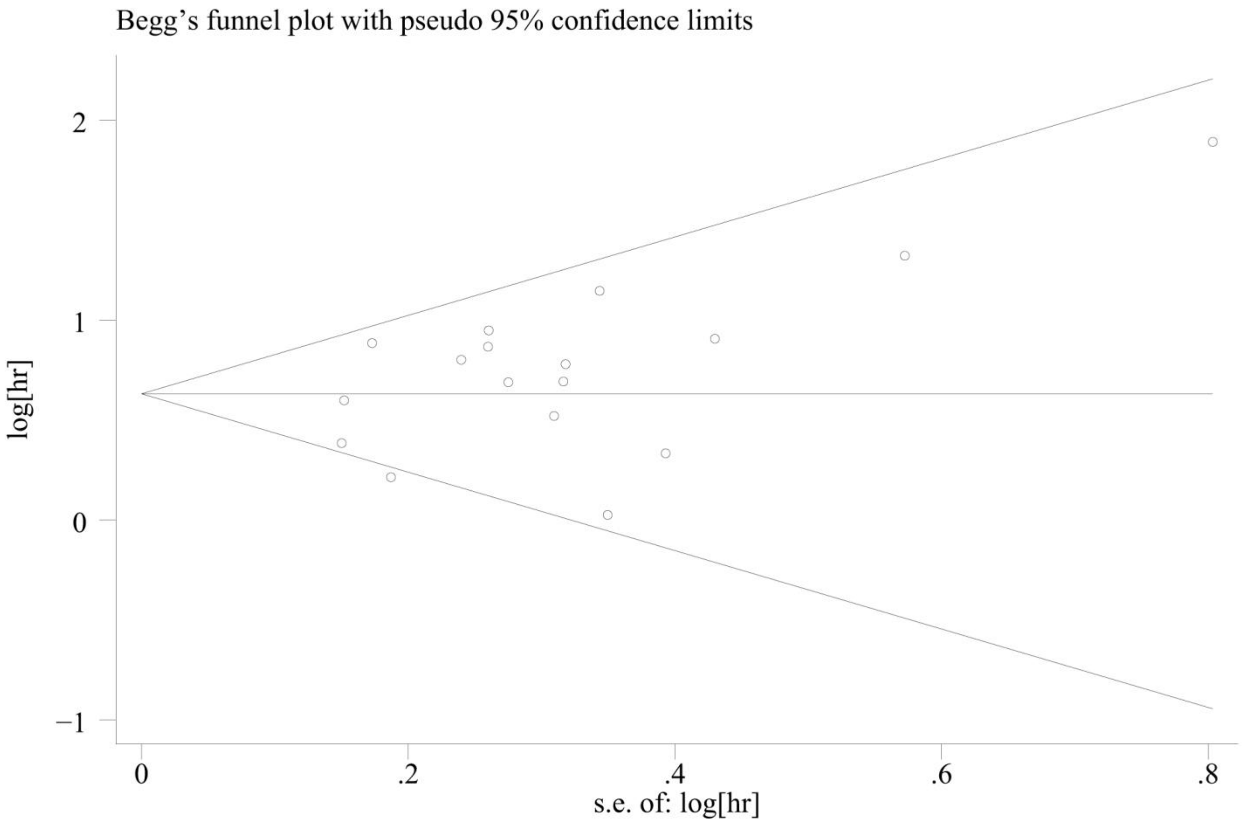

Supplementary Figure 2c. Begg's funnel plot of OS in RCC patients treated with targeted therapy with high NEU versus low NEU.

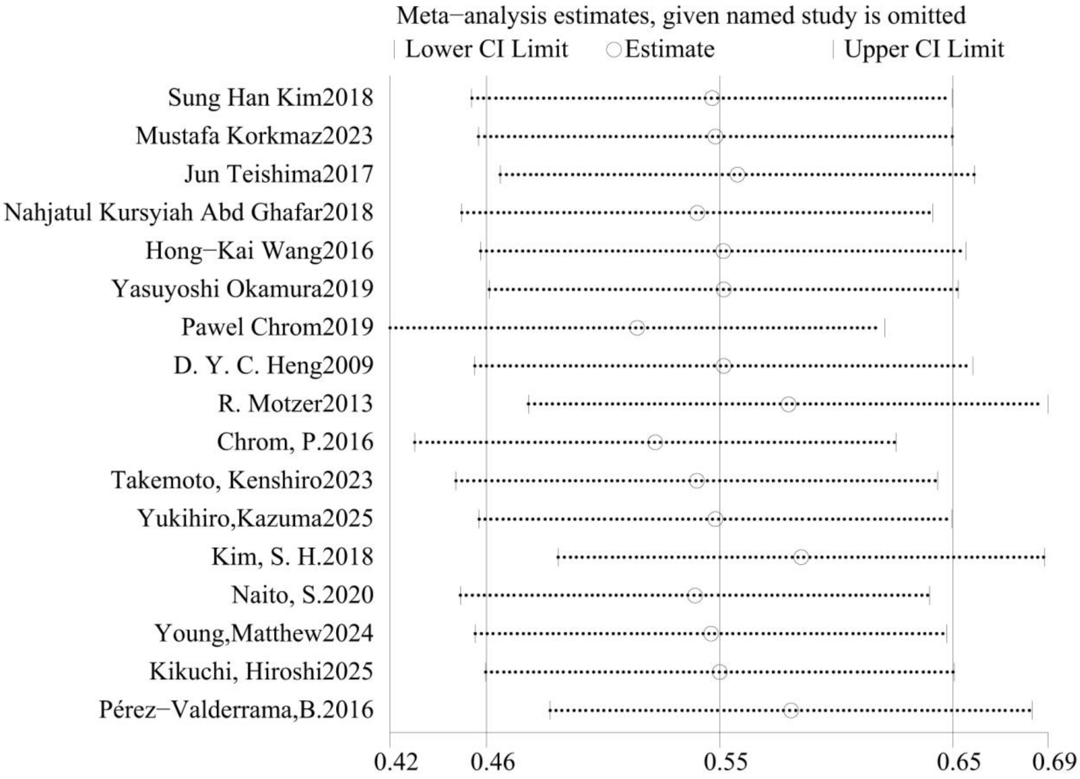

Supplementary Figure 3a. Sensitivity analysis of OS in RCC patients treated with targeted therapy with low Hb versus high Hb.

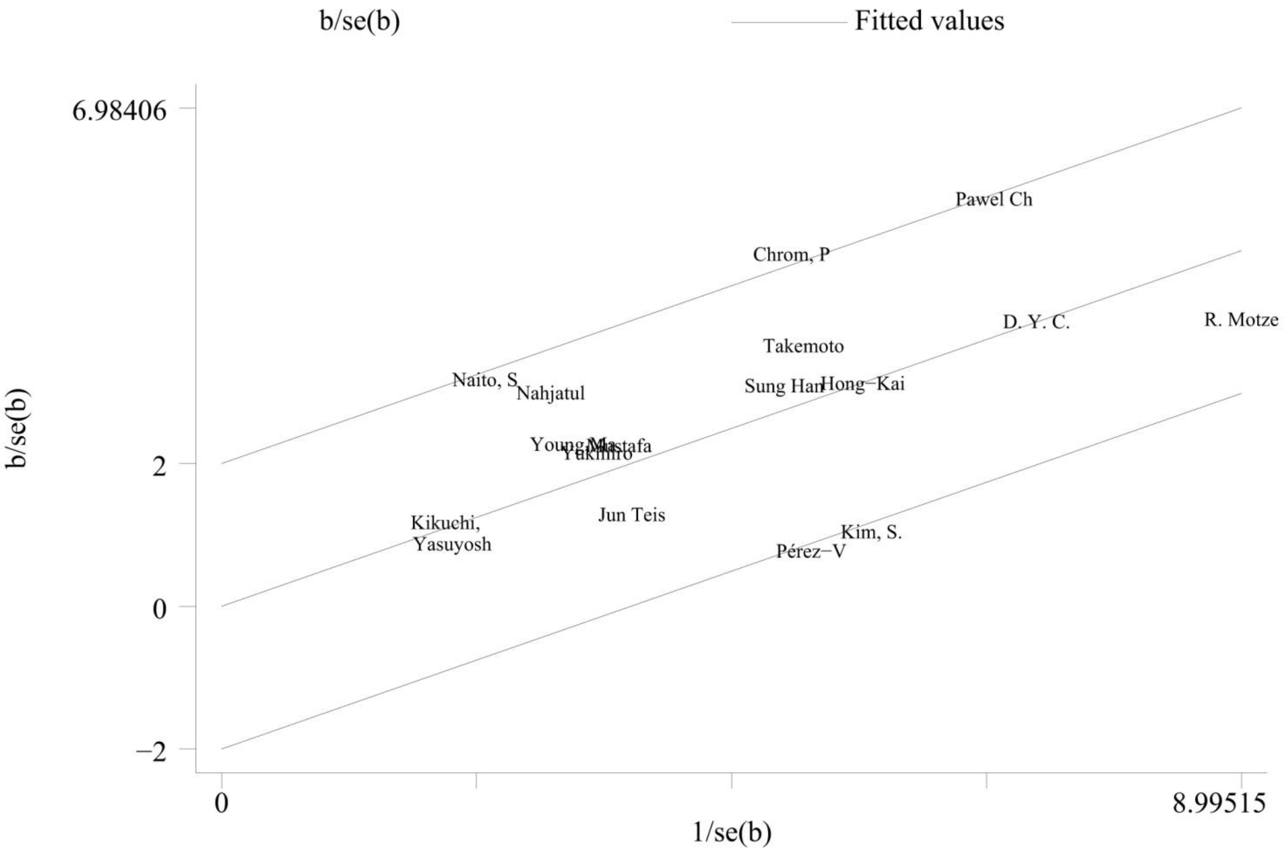

Supplementary Figure 3b. Galbraith plot of OS in RCC patients treated with targeted therapy with low Hb versus high Hb.

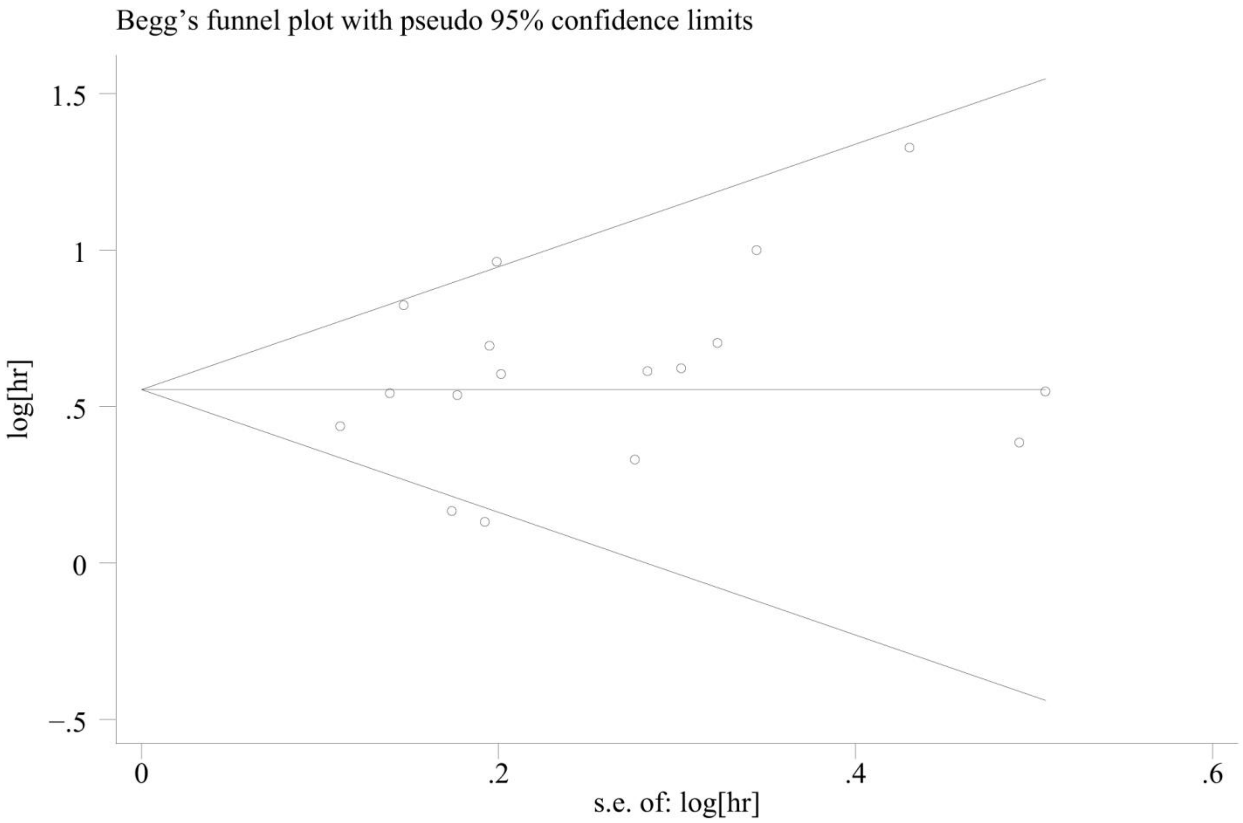

Supplementary Figure 3c. Begg's funnel plot of OS in RCC patients treated with targeted therapy with low Hb versus high Hb.

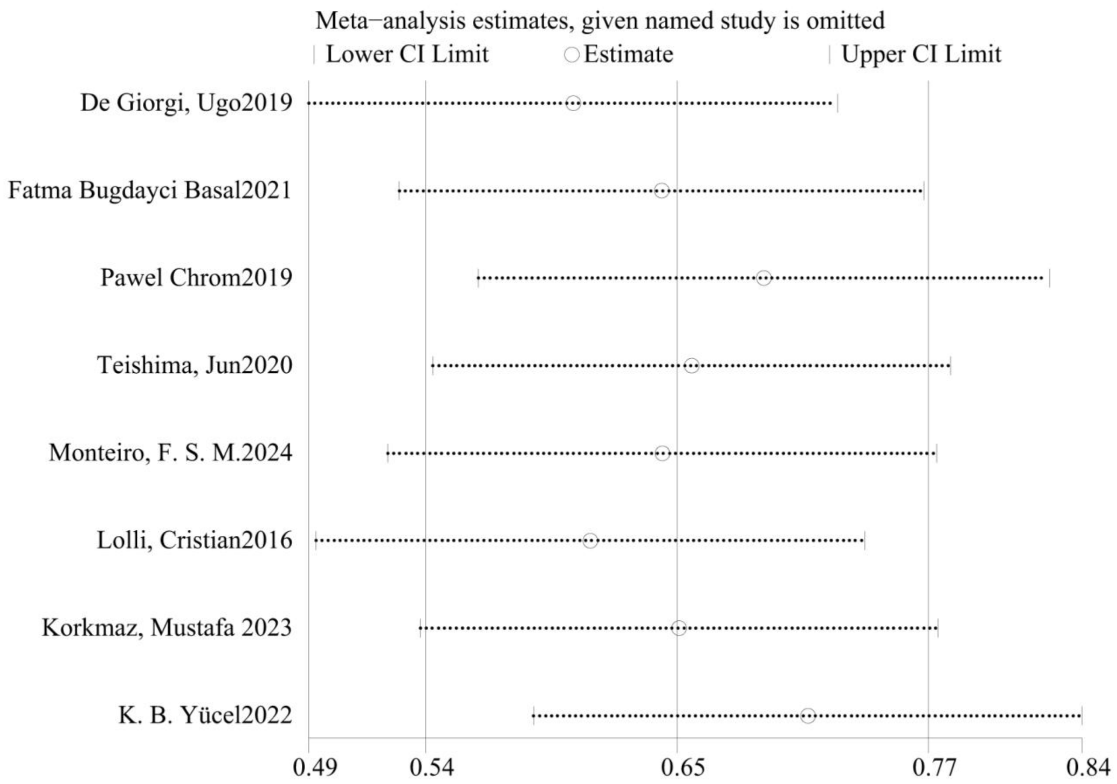

Supplementary Figure 4a. Sensitivity analysis of OS in RCC patients treated with targeted therapy with high SII versus low SII.

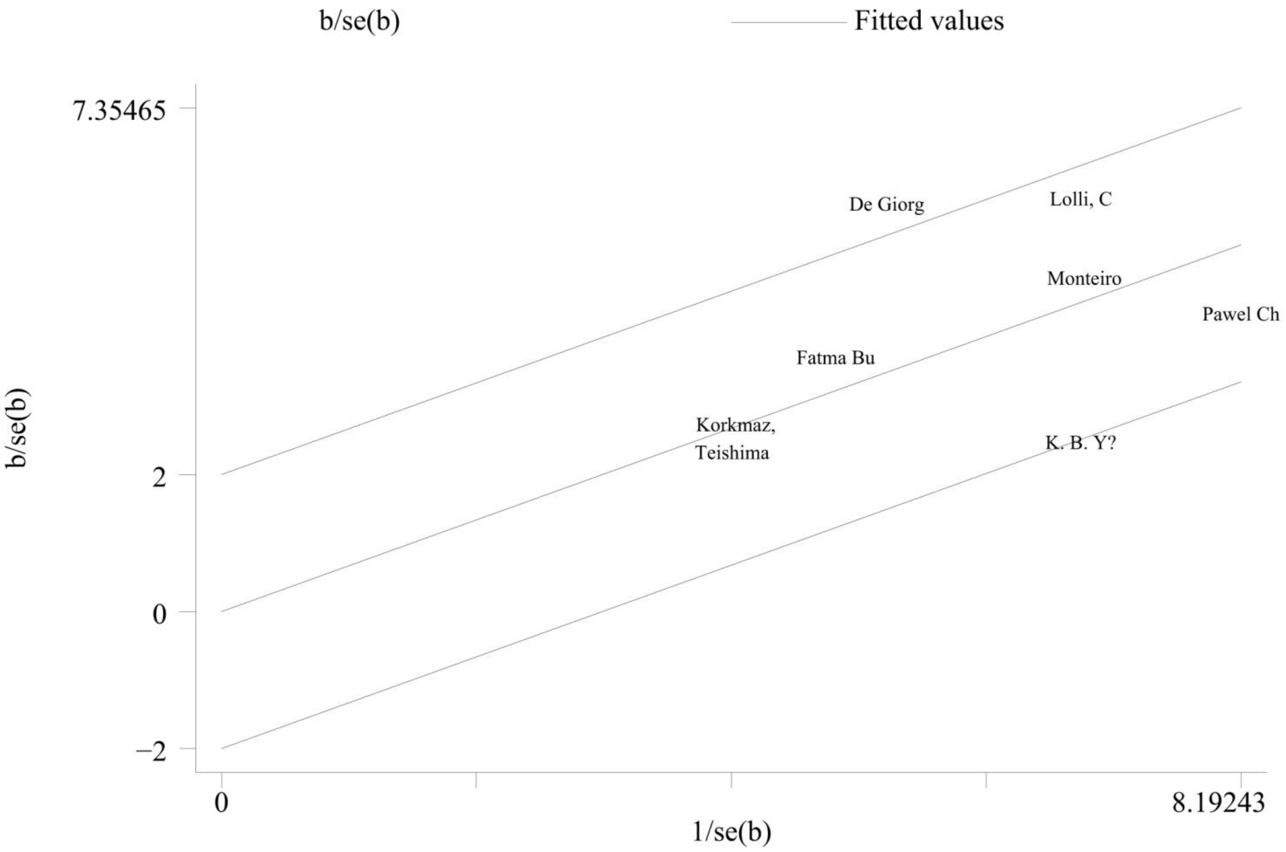

Supplementary Figure 4b. Galbraith plot of OS in RCC patients treated with targeted therapy with high SII versus low SII.

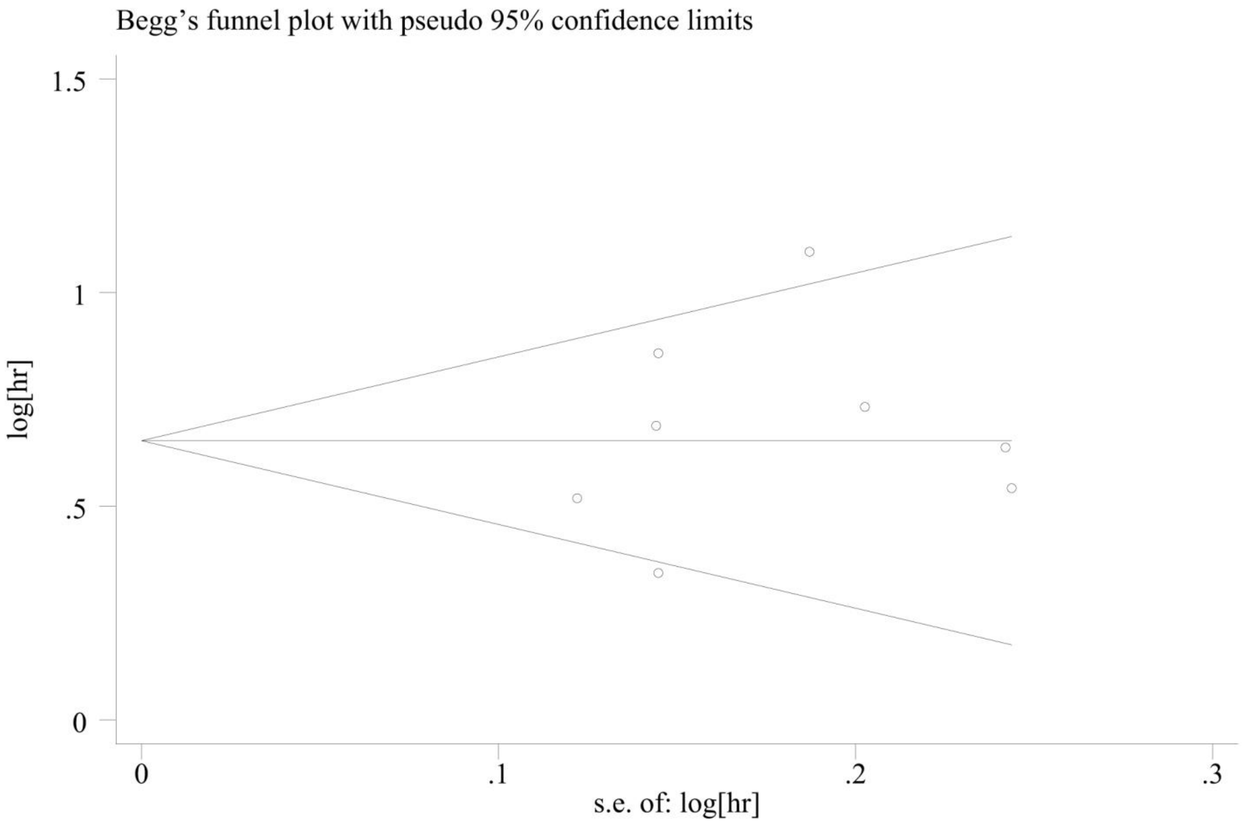

Supplementary Figure 4c. Begg's funnel plot of OS in RCC patients treated with targeted therapy with high SII versus low SII.
